# Drugging the Undruggable in Oncology: Structural Computation, Machine Learning, and Generative AI for Historically Intractable Targets

**DOI:** 10.3390/ph19101578

**Published:** 2026-10-05

**Authors:** Mikołaj Stańczak, Sarfaraz Niazi

**Affiliations:** 1 School of Mathematical and Computer Science, Heriot-Watt University, Edinburgh EH14 4AS, UK; 2 College of Pharmacy and Pharmaceutical Sciences, Washington State University, Spokane, WA 99202, USA; s.niazi@wsu.edu

**Keywords:** undruggable targets, KRAS, p53, MYC, SHP2, generative chemistry, equivariant neural networks, diffusion models, free-energy perturbation, AlphaFold, cryptic pockets, molecular glues, targeted protein degradation, covalent inhibition, pan-cancer drug discovery

## Abstract

For four decades the central oncogenic drivers of human cancer the RAS GTPases, the transcription-factor MYC, mutant p53, and the protein-tyrosine phosphatases, fusion oncoproteins and intrinsically disordered regulators that surround them were regarded as undruggable: lacking deep, well-defined small-molecule binding pockets, governed by femtomolar substrate affinities, or driven through flat protein–protein interfaces that classical medicinal chemistry could not engage. The last decade has overturned that verdict. This narrative review argues that the convergence of three advances has made the undruggable proteome systematically approachable: the discovery of cryptic, allosteric and induced pockets through molecular dynamics and deep learning; the maturation of physics-based and machine-learned models that can read out binding from structure; and the rise in generative artificial intelligence that can design novel molecules conditioned directly on a three-dimensional target. We develop the argument in full formal detail. We set out the mathematics of modern molecular machine learning E(3)/SE(3)-equivariant graph neural networks, denoising diffusion and score-based generative models, and generative flow networks alongside the statistical mechanics of binding free energy (free-energy perturbation, thermodynamic integration, MM-PBSA), the kinetics of covalent inhibition, the equilibrium theory of ternary complexes and cooperativity that governs molecular glues and degraders, and the pharmacology that determines whether a designed molecule reaches and acts on a tumour. We then examine, target by target, the biology that made each oncoprotein intractable and the campaigns structural, chemical and increasingly computational that have begun to defeat it, distinguishing throughout what classical structural and computational work achieved, what machine learning demonstrably contributed, and what generative design has so far only promised: the switch-II pocket and the covalent KRAS-G12C and non-covalent KRAS-G12D and pan-RAS(ON) inhibitors; the mutation-created cavity of p53-Y220C and its first reactivators; the disordered MYC-MAX interface and the miniprotein Omomyc; and the allosteric tunnel of SHP2. These four are selected for mechanistic non-redundancy, on grounds we state explicitly, and the structured search framework and eligibility criteria underlying the review are documented. We foreground the newest evidence AlphaFold3, RoseTTAFold All-Atom, deep cryptic-pocket predictors, and the first generative-to-clinic existence proofs while maintaining candour about the field’s hard limits: the physical-validity and generalisation gaps of deep docking, the bounded accuracy and cost of free-energy methods, the synthesizability gap, dual-use risk, and a clinical attrition that artificial intelligence has not yet reduced. The synthesis we offer is not that the problem is solved but that it has changed in kind: from a search for the rare tractable target to the deliberate, computable engineering of molecules against targets once thought beyond reach.

## 1. Introduction

### 1.1. The Undruggable Proteome

The molecular drivers of human cancer have long been divided into two populations of very unequal therapeutic fortune. On one side stand the kinases, nuclear receptors and a handful of enzymes whose deep, hydrophobic, well-defined active sites accommodate drug-like small molecules; on the other stand the proteins responsible for the largest share of oncogenic signalling the RAS GTPases, the transcription-factor MYC, the tumour suppressor p53 in its mutant forms, the protein-tyrosine phosphatases, and the fusion oncoproteins and intrinsically disordered regulators that pervade the cancer genome which for decades resisted every attempt at small-molecule inhibition and were accordingly labelled undruggable [1,2]. The label was never a statement about biological importance, which is in these cases supreme, but about tractability: these proteins present shallow or featureless surfaces, bind their physiological partners across broad protein–protein interfaces, or, in the case of RAS, engage their nucleotide substrate with picomolar-to-femtomolar affinity against a cellular pool of millimolar GTP, leaving no thermodynamic foothold for a competitive ligand [3].

Three structural features recur across the undruggable class and explain the failure of classical approaches. First, the absence of a deep orthosteric pocket: transcription factors such as MYC function through an extended, largely disordered interface with MAX rather than through a compact catalytic site, so there is no cavity to fill [4,5]. Second, the dominance of protein–protein interactions, which bury large, flat, polar surface areas that small molecules engage only weakly and non-selectively [6]. Third, extreme substrate competition, exemplified by the RAS GTPase cycle, where any active-site ligand must outcompete a vast excess of high-affinity nucleotide. To these one may add the problem of selectivity within large paralogous families, acute for the phosphatases, and the conformational heterogeneity of intrinsically disordered regions, which present not one structure to be targeted but a fluctuating ensemble [5]. Against this backdrop, the pharmaceutical industry concentrated, rationally, on the tractable minority, and the central oncogenes remained beyond reach.

### 1.2. Why the Verdict Is Changing

That situation has been overturned within a little more than a decade, and this review argues that the reversal is the joint product of three advances, each of which artificial intelligence and physics-based computation have sharply accelerated. The first is a change in our conception of a binding site. High-resolution molecular dynamics, enhanced sampling and, most recently, deep learning have shown that many proteins lacking a pocket in their ground-state crystal structure transiently open cryptic or allosteric pockets, and that such sites can be predicted, induced and exploited [7,8]. The paradigmatic case is the KRAS switch-II pocket, invisible in the canonical structure yet revealed by a covalent ligand, whose discovery converted the most famous undruggable target into a clinically validated one [9]. The second advance is the maturation of methods that read out binding affinity from three-dimensional structure: rigorous free-energy calculations now reach roughly chemical accuracy in favourable regimes, and machine-learned scoring functions, for all their limitations, allow billions of molecules to be triaged computationally [10,11]. The third, and the organising theme of this review, is generative artificial intelligence: models that do not merely screen existing libraries but design novel molecules conditioned directly on a protein structure, on a pocket, or on a desired property profile [12,13].

The convergence matters because the undruggable problem is fundamentally one of finding, and then filling, the rare and often transient cavities on otherwise intractable surfaces, and of doing so with molecules that may need to be covalent, allosteric, bifunctional, or capable of acting on a conformational ensemble rather than a single structure. Each of these requirements maps onto a computational capability that has only recently become reliable. The remainder of this review develops that mapping in detail, but its thesis can be stated at the outset: the druggability of a target is not a fixed property but a function of the methods brought to bear on it, and the methods have changed.

Three developments since 2024 have sharpened each of these claims, and they are worth stating at the outset, because the introduction to a review of this kind should rest on the current state of the art rather than on the benchmark surveys that defined the problem a decade ago. The first is that structure prediction has moved from single chains to biomolecular complexes: AlphaFold3 and RoseTTAFold All-Atom predict protein–ligand, protein-nucleic-acid and covalently modified assemblies within a single framework [14,15], and a family of open reimplementations Boltz-1, Chai-1 and the affinity-predicting Boltz-2 has made that capability broadly available and, as importantly, independently testable [16,17,18]. The second is that independent evaluation has begun to discipline the claims made for these models: a systematic assessment on structures released after the training cut-off found that co-folding accuracy depends strongly on similarity to the training set, and concluded that current methods largely reproduce ligand poses encountered in training rather than generalising to genuinely novel chemistry [19]. The third is that the proximity-inducing modalities have moved from mechanism to medicine: the chemical space and design principles of molecular glues have begun to be systematised [20], the first targeted protein degrader to show a phase 3 benefit has since become an approved medicine, the FDA having licenced vepdegestrant in May 2026 for ESR1-mutated, oestrogen-receptor-positive, HER2-negative advanced or metastatic breast cancer after at least one prior line of endocrine therapy [21,22,23], and in RAS the mutation-agnostic tri-complex strategy has produced a randomised survival benefit in pancreatic cancer [24]. Taken together, these three developments define the terrain this review surveys: capabilities that are real and improving quickly, claims that require independent verification before they are believed, and a modality landscape that has enlarged faster than the computational methods that would design for it.

### 1.3. Scope, Approach, and Structure

This is a narrative review, and the form is chosen deliberately. The subject spans four largely separate literatures machine learning, structural biology and biophysics, the medicinal chemistry of novel modalities, and the molecular and cellular biology of oncogenes and the contribution we aim to make is integrative rather than enumerative: to show how advances in each domain combine to render the undruggable approachable, and to do so with the formal apparatus intact. We therefore foreground the newest work, from AlphaFold3 and RoseTTAFold All-Atom to deep cryptic-pocket predictors and the first generative-to-clinic existence proofs, while grounding each theme in its landmark antecedents [25,26]. Consistent with the narrative form, we do not claim a systematic survey of every method or target, and we are explicit throughout about where evidence is strong, where it is preliminary, and where it is contested.

**Figure 1:**
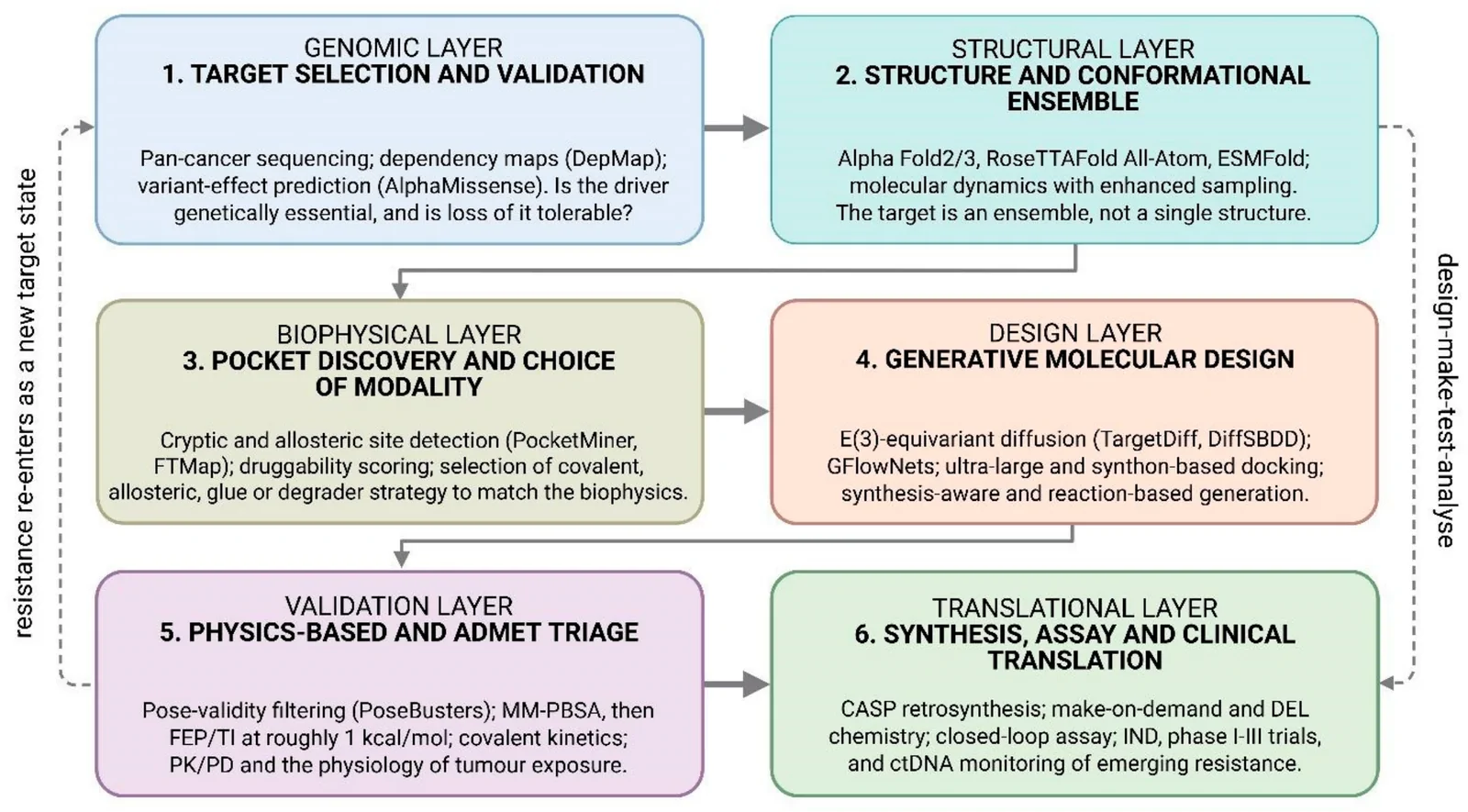
The integrated AI-driven design pipeline, from the genomic level to clinical application. Solid arrows trace the forward workflow; dashed arrows are the two feedback loops that make the pipeline iterative rather than linear. Each layer has a characteristic strength and a characteristic failure mode, and the competence of the whole is limited by its weakest link. The layers correspond to Section 3.4 and Section 5 (target), Section 3 (structure and ensemble), Section 3.3 and Section 6 (pocket and modality), Section 2 (generative design), Section 4 (physics-based evaluation), Section 2.6 and Section 7 (synthesis and translation). Diagrammatic; not drawn to scale. Created in BioRender. Niazi, S. (2026) https://BioRender.com/4nrdcxj.

The review proceeds as follows. Section 1.4 and Section 1.5 first report how the literature was identified and set out the grounds on which the four detailed targets were chosen. Section 2 then sets out the mathematics of modern molecular machine learning. Section 3 treats protein structure prediction and the computational discovery of cryptic and allosteric pockets that expand the druggable proteome. Section 4 develops the physics and biochemistry of binding free-energy methods, molecular dynamics, covalent and binding kinetics, and the pharmacology of tumour exposure with their governing equations. Section 5 turns to the targets themselves, examining KRAS, p53, MYC and SHP2, the biology that made each intractable and the campaigns that have begun to defeat it. Section 6 surveys the modalities covalent, allosteric, molecular-glue and degrader, and polypharmacological that expand the space of what a small molecule can do, with the equilibrium and kinetic theory that governs them. Section 7 assesses translation and the existence proofs, Section 8 the safety and dual-use considerations, and Section 9 and Section 10 the synthesis and conclusions. The integrated and iterative workflow connecting these stages, from target selection and structural modelling to molecular design, physics-based evaluation and clinical translation, is summarized in Figure 1.

One distinction runs through everything that follows, and it is worth making explicit at the outset because the literature routinely blurs it. When we say that a target has yielded to computation, three quite different kinds of claim are available, and they carry very different evidential weight. The first concerns the experimental, structural and classical computational work crystallography and cryo-electron microscopy, fragment and covalent screening, molecular dynamics, docking and conventional structure-based optimisation that actually opened each of these targets, and which in every case described in Section 5 did the decisive work. The second concerns demonstrated contributions of machine learning, in which a learned model measurably assisted the discovery or optimisation of a molecule that exists. The third concerns the prospective role of generative artificial intelligence: what these methods plausibly could contribute, argued from their formal properties and from results in adjacent problems, but which they have not yet contributed to any approved or clinically successful agent against the four targets treated here.

We keep these three categories apart deliberately, and label them where the distinction could otherwise be lost, including in Table 1. The reason is not modesty but consistency. Section 7 reports candidly that the prospective generative design of a small molecule against a genuinely novel undruggable oncology target, carried through to the clinic, has not yet been demonstrated; an introduction that implied otherwise would be at odds with the paper’s own conclusion. The history of these four targets is a history of structural biology and medicinal chemistry, assisted increasingly by computation. The argument of this review is that the newer methods change what is now attemptable, not that they already account for what has been achieved.

### 1.4. Literature Search Strategy

Although this is a narrative rather than a systematic review, and makes no claim to exhaustive coverage, the literature underpinning it was identified through a structured search, which we document here so that readers can judge its scope and its limits. Searches were run in PubMed/MEDLINE, Scopus and the Web of Science Core Collection for the peer-reviewed biomedical and chemical literature, and in arXiv, bioRxiv and ChemRxiv together with the proceedings of NeurIPS, ICML and ICLR for the machine-learning literature, a substantial part of which in this field never appears in journals at all. ClinicalTrials.gov was searched for trial status, and the abstract archives of AACR, ASCO and ESMO for clinical results not yet in full publication. The search covered 1 January 2013 to 31 May 2026 and was last updated in May 2026, with no lower date limit applied to the landmark methodological and biological papers reached by backward citation chaining. Clinical, regulatory and bibliographic status was then refreshed through 31 August 2026, so that the agents, approvals and trial readouts discussed below are current at the time of writing rather than at the close of the literature search.

Terms were combined in three blocks joined by the Boolean AND, with terms within each block joined by OR: a target block, a method block and a modality block, set out in full in Table 2. Records were screened at title and abstract for relevance to small-molecule design against the target classes considered here; the reference lists of retrieved reviews were then chained backwards, and forward citation searching was used to capture work published after each landmark. We included primary methodological papers, structural and biophysical studies and clinical reports, and used reviews for context rather than as evidence. We excluded non-English records and abstracts reporting no data, and we cited preprints only where no peer-reviewed version exists, marking them as such. Two limitations should be stated plainly rather than left to be inferred. There was no PRISMA flow diagram, no dual independent screening and no formal risk-of-bias assessment, none of which a narrative review spanning four disparate literatures can honestly claim; and the selection of material is therefore interpretive, which is precisely why the text is explicit throughout about where the evidence is strong, where it is preliminary and where it is contested. We also make no claim that these searches are reproducible in the strict sense. A fully reproducible electronic search would require database-specific strings, exact search dates, retrieval counts and a description of deduplication, none of which we recorded prospectively; what we document is the structured framework and the eligibility criteria, which is the appropriate standard for a narrative review and should not be mistaken for a systematic one.

### 1.5. Target Selection: Rationale and Boundaries

A review that treats four proteins in depth owes an account of why those four and not others of comparable importance. The selection followed three criteria, applied deliberately rather than by convenience, and Table 3 records the resulting decisions.

The first criterion is mechanistic distinctness. The purpose of this review is to show how a computational method maps onto a biophysical obstacle, and that mapping is only visible if the obstacles differ. KRAS presents overwhelming substrate competition and a pocket that does not exist at rest. p53 presents not an excess of function to be blocked but a loss of function to be restored, together with a cavity created by the mutation itself. MYC presents an extended, intrinsically disordered interface with no folded pocket at all. SHP2 presents a catalytic site too polar and too conserved to drug selectively, and an allosteric solution remote from it. Four targets, four distinct failure modes of classical medicinal chemistry, and four distinct computational responses: cryptic-pocket detection coupled to covalent chemistry, conformational stabilisation, ensemble-based design against disorder, and allosteric modulation. A fifth target sharing a mechanism already covered would have added length without adding argument.

The second criterion is evidentiary depth. Each of the four supports a campaign that can be traced from structural hypothesis through chemical matter to clinical test, which is what makes them useful as worked examples rather than as illustrations. Approved drugs exist for KRAS-G12C, and clinical agents for p53-Y220C, MYC and SHP2; the computational contribution at each stage is documented; and which matters at least as much for a review intending to be candid so are the failures.

The third criterion is boundary clarity, and it requires naming what was excluded. STAT3 and the β-catenin/WNT effectors share the transcription-factor and interface problem with MYC, and the arguments developed for MYC in Section 5.3 apply to them with little modification; they are therefore treated by generalisation in Section 5.5. The BCL-2 family is, by the standard used here, a solved rather than an open problem: the fragment-based, structure-guided campaign that produced venetoclax was conducted largely before the generative era, and is better read as the precedent that established that protein–protein interfaces contain druggable hot spots at all than as a current frontier. The non-KRAS RAS isoforms share the biophysics of KRAS almost exactly, so that treating them separately would duplicate Section 5.1; isoform-specific considerations are noted there. Epigenetic reader and writer domains possess well-formed, conventionally druggable pockets, and their difficulties have been those of selectivity, on-target toxicity and therapeutic index rather than of tractability; they were never members of the undruggable class as it was defined in Section 1.1. Undruggability, in short, was the inclusion criterion, and mechanistic non-redundancy the tie-breaker.

## 2. Mathematical Foundations of Molecular Machine Learning

A note on how to use this section. The mathematics that follows is set out in full because the argument of this review depends on it: claims about what generative models can and cannot do against an undruggable target are claims about the structure of those models, and cannot be assessed if the models are treated as black boxes. But the formalism is a means rather than the point, and a reader coming from medicinal chemistry or cancer biology should not have to absorb equivariance and stochastic differential equations before learning why either matters. We therefore state the problem first, in plain terms, and the machinery afterwards.

The problem is this. A molecule capable of binding a shallow, transient pocket on an undruggable protein almost certainly does not exist in any catalogue; it has to be invented. Inventing it requires a system that can do four things. It must represent a molecule in a way that retains the three-dimensional information determining whether it fits a cavity, rather than only its connectivity. It must propose new molecules, not merely score existing ones. It must propose many different good molecules rather than converging on a single optimum, because any one chemotype will usually fail for reasons of synthesis, selectivity or pharmacokinetics that have nothing to do with binding. And its proposals must be checkable, because a molecule that satisfies a model may be physically impossible or synthetically inaccessible. Each of the subsections that follow answers one of these requirements in turn, and Table 4 states the correspondence explicitly, together with the characteristic way in which each method fails. A reader whose interest is principally biological may take the table and proceed to Section 3; the derivations will still be here.

### 2.1. Representing Molecules: Strings, Graphs, and Three-Dimensional Point Clouds

A learning algorithm can only be as good as the representation on which it operates, and the history of molecular machine learning is in large part a history of increasingly faithful representations. The earliest and still most widely used is the molecular string, of which SMILES is canonical: a molecule is linearised into a sequence of tokens encoding atoms, bonds and ring closures, after which the full machinery of sequence models becomes applicable [27]. Strings are compact and enable powerful generative language models, but a single molecule admits many valid strings and small edits can yield invalid chemistry; SELFIES was introduced precisely to guarantee that every string decodes to a valid molecule, a property that matters greatly for generative search [28]. The string representation underpinned the first wave of deep generative chemistry: continuous latent-variable models that embed molecules in a smooth space amenable to optimisation [29], recurrent language models that learn the grammar of SMILES and can be focused toward desired properties by transfer learning [30], and reinforcement-learning agents that fine-tune such generators against a scoring function to bias sampling toward active chemotypes [31] the conceptual ancestors of the structure-conditioned models developed below.

A more natural representation treats a molecule as a graph G = (V, E), with atoms as nodes carrying feature vectors and bonds as edges. Graph neural networks operate on this structure by iteratively passing messages between neighbouring nodes and updating node states, a scheme formalised as the message-passing neural network [32,33]. For a node i with neighbourhood N(i), one round of message passing computes an aggregated message and a state update, written schematically as

*m_i =* *Σ_{j* *∈* *N(i)} M(h_i, h_j, e_{ij}), h_i′* *= U(h_i, m_i)*

where M and U are learned functions and e_{ij} encodes bond features. Graph representations capture connectivity and chemistry but discard three-dimensional geometry, which is precisely what determines whether a molecule fits a binding pocket. The frontier representation is therefore the three-dimensional point cloud: each atom carries both invariant features h_i and a coordinate x_i ∈ ℝ^3^, and the model must respect the symmetries of physical space. It is the demand for geometric symmetry that gives rise to the equivariant networks at the heart of modern structure-based design.

### 2.2. Geometric Deep Learning and Equivariance

The physics of binding is indifferent to the absolute position and orientation of a molecule in space: rotating or translating a protein–ligand complex leaves its energy unchanged. A model that predicts geometric quantities should encode this symmetry exactly rather than learn it approximately from data, and the framework that formalises this requirement is geometric deep learning [34]. A function f is said to be equivariant to a group G of transformations if applying a transformation to the input produces the correspondingly transformed output. For the Euclidean group acting on coordinates, the condition is

*f(Q x + t) = Q f(x) + t, for all Q* *∈* *SO(3), t* *∈ ℝ*^3^

for equivariant (vector-valued) outputs such as coordinates, while scalar outputs such as energies must be invariant, f(Q x + t) = f(x). A terminological distinction matters here and is frequently blurred. SE(3) is the special Euclidean group of proper rotations and translations; E(3) additionally admits reflections, so a model described as E(n)-equivariant makes the stronger claim, and for chiral molecules that extra invariance must be handled deliberately, since enantiomers are not pharmacologically interchangeable. Early approaches achieved rotational equivariance using bases of spherical harmonics and irreducible representations of SO(3), as in tensor field networks and the SE(3)-Transformer, which are expressive but computationally heavy [35,36,37]. A decisive simplification was the E(n)-equivariant graph neural network (EGNN), which attains equivariance without spherical harmonics by making messages depend only on quantities that are themselves invariant chiefly the squared interatomic distance and by updating coordinates strictly along relative position vectors [38]. Concretely, for nodes carrying invariant features h_i and coordinates x_i, one EGNN layer computes

*m_{ij} =* *φ_e(h_i^l, h_j^l, ‖ x_i^l − x_j^l ‖*^2^*, e_{ij})*

*x_i^{l + 1} = x_i^l +* *Σ_{j≠i} (x_i^l − x_j^l) ·* *φ_x(m_{ij})*

*h_i^{l + 1} =* *φ_h(h_i^l,* *Σ_{j≠i} m_{ij})*

Because the message φ_e depends on the pair only through the rotation-invariant distance, and because each coordinate is displaced along the relative vector (x_i − x_j), which rotates with the input, the layer is provably E(n)-equivariant: rotate or translate the input and the updated coordinates transform identically while the features are unchanged [38]. This construction is the workhorse of contemporary three-dimensional molecular generation. Recent extensions augment it with virtual nodes to improve the identification of binding sites, illustrating how the basic equivariant primitive continues to be refined for structure-based tasks [39]. The same inductive bias build in the symmetry rather than learn it underlies the geometric and equivariant transformer architectures now widely used for three-dimensional molecular modelling. Ordinary transformer attention carries no intrinsic geometric equivariance; where such architectures respect rotation and translation, the symmetry is supplied by the representation or built into the attention mechanism rather than inherited from the transformer itself [40].

### 2.3. Denoising Diffusion and Score-Based Generative Models

The generative engine that has come to dominate structure-based design is the diffusion model, introduced from non-equilibrium thermodynamics and developed into the denoising diffusion probabilistic model (DDPM) [41,42]. The idea is to define a fixed forward process that gradually corrupts data into noise, then to learn the reverse process that reconstructs data from noise. Given a data point z_0 and a variance schedule {β_t} for t = 1, …, T, the forward process adds Gaussian noise at each step,

*q(z_t | z_{t−1}) =*
 *𝒩*
 *(z_t; √(1−β_t) · z_{t−1}, β_t I)*

and, defining α_t = 1 − β_t and the cumulative product Ā_t = Π_{s = 1}^{t} α_s, admits the closed-form marginal that allows sampling any noise level directly,

*z_t = √(Ā_t) · z_0 + √(1 − Ā_t) · ε, ε ~*
 *𝒩*
 *(0, I)*

A neural network ε_θ is trained to predict the noise added at each step by minimising a simple reweighted objective, after which data are generated by iterating the learned reverse step from pure noise,

*z_{t−1} = (1/√α_t) (z_t − (β_t/√(1−Ā_t)) · ε_θ(z_t, t)) + σ_t ξ, ξ ~*
 *𝒩*
 *(0, I)*

A unifying continuous-time formulation casts both forward and reverse processes as stochastic differential equations [43]. The forward corruption is dx = f(x,t) dt + g(t) dw, and its exact time reversal, which generates data, is

$$d_{x}=[f(x,t)-g(t{)}^{2}\;\nabla \_x\;log\;p\_t(x)]\;dt+g(t)\;d\bar{w}$$

where $d\bar{w}$ is a reverse-time Wiener increment and ∇_x log p_t(x) is the score, the gradient of the log-density, which the network learns by denoising score matching. The deterministic probability-flow ordinary differential equation associated with this SDE enables fast, exact-likelihood sampling. For molecular generation, the data are atomic coordinates and types, and the denoising network is an equivariant graph network of the kind described above, so that the entire generative process respects Euclidean symmetry [44]. The step from unconditional generation to structure-based drug design is then conceptually direct: one conditions the reverse process on the protein pocket C, learning ε_θ(z_t^L, t, C) so that the ligand atoms L are denoised in the context of the target. Pocket-conditioned models including Pocket2Mol, TargetDiff and DiffSBDD instantiate exactly this scheme, generating three-dimensional molecules inside a binding site in a single non-autoregressive process [12,13,45]. Because the noise distribution can be sampled indefinitely, such models propose unlimited novel candidates geometrically complementary to a target a capability of obvious value when the target is one for which no inhibitor chemotype previously existed.

### 2.4. Generative Flow Networks

A complementary generative paradigm addresses a limitation of reward-maximising approaches: when a molecule is built up through a sequence of discrete actions and scored by some reward R(x), reinforcement learning tends to collapse onto a few high-reward modes, whereas drug discovery benefits from many diverse high-reward candidates. Generative flow networks (GFlowNets) are designed to sample compositional objects with probability proportional to their reward rather than to maximise it [46]. Treating molecule construction as a directed acyclic graph of states s and actions, one learns a forward policy P_F and backward policy P_B and a scalar partition function Z, optimised by the trajectory-balance objective over complete trajectories τ,

*ℒ_TB(τ) = (log [Z_θ · Π_t P_F(s_{t + 1} | s_t)] − log [R(x) · Π_t P_B(s_{t−1} | s_t)])*^2^

At the optimum, the learned sampler satisfies the defining proportionality π(x) ∝ R(x), with the normalisation Z = Σ_x R(x), so that the network draws candidates across the full landscape of acceptable molecules rather than from a single peak [46,47]. Trajectory balance markedly improves credit assignment and convergence relative to earlier flow-matching objectives and yields more diverse candidate sets, as measured by lower pairwise similarity among top-scoring molecules [48]. For undruggable targets, where chemical novelty is at a premium, and the optimal scaffold is unknown a priori, the explicit pursuit of diversity is a meaningful advantage over methods that converge prematurely.

### 2.5. Docking, Machine-Learned Scoring, and the Validity Gap

Generation must be paired with evaluation, and the fast end of the evaluation spectrum is molecular docking: predicting the bound pose of a ligand in a pocket and estimating its affinity with an empirical or knowledge-based scoring function. The classical tools AutoDock Vina, Glide, and GNINA, the last incorporating a learned convolutional scorer remain the workhorses of virtual screening and now support docking campaigns over libraries of billions of make-on-demand compounds [11,49,50,51]. Deep learning has been brought to docking itself: DiffDock reframes blind docking as a diffusion process over the manifold of ligand poses, parameterised by translational, rotational and torsional degrees of freedom, and reports strong top-pose accuracy by root-mean-square deviation [52].

Yet a sobering and important corrective has emerged. The PoseBusters analysis showed that when poses are checked for physical validity correct bond lengths and angles, planar aromatic rings, the absence of steric clashes with the protein deep-learning docking methods frequently fail, and classical physics-based methods such as Vina and GOLD outperform them; moreover, the deep methods generalise poorly to protein sequences dissimilar from their training set [53]. A favourable RMSD, in other words, does not guarantee a physically realisable complex. The same theme of inflated benchmark performance recurs across molecular machine learning: standardised suites such as GuacaMol and MOSES make generative models comparable but optimise proxy objectives rather than therapeutic utility, and ligand-based classification benchmarks have been shown to reward memorisation of training scaffolds rather than genuine generalisation [54,55,56]. Influential commentary has warned that generative models often propose molecules that are attractive on paper yet hard to synthesise or inactive in assay, and that the gap between in silico promise and validated chemical matter must be discounted accordingly [57,58]. These critiques do not negate the power of the methods; they define the discipline required to use them, and they motivate the layered architecture fast generation and scoring, then rigorous physics, then experiment that recurs throughout this review. The newest agentic systems, which couple large language models to chemistry tools and databases to plan and execute tasks, extend rather than escape this requirement for physical and experimental grounding [59]. A parallel response to the opacity of these models has been the development of explainable approaches, which seek not merely a prediction but an interpretable rationale for it, so that a chemist can judge whether a model has learned genuine structure-activity relationships or a spurious correlation a concern of particular weight when designing against a novel target for which intuition offers little check [60].

### 2.6. Closing the Synthesizability Gap: Computational Strategies

Section 2.5 recorded the standing criticism that generative models propose molecules that are attractive on paper and hard or impossible to make [57,58]. Recording that failure is not sufficient, because the gap is now addressable, and the methods that address it belong in any serious account of generative design. They fall into three families of increasing strength, and the distinction matters practically, because they are not interchangeable.

The weakest, and by some distance the most widely used, are post hoc scores applied to a molecule after it has been generated. The synthetic accessibility score of Ertl and Schuffenhauer estimates ease of synthesis from fragment contributions calibrated on a large corpus of known compounds, together with a penalty for molecular complexity [61]. SCScore learns synthetic complexity directly from a reaction corpus, on the principle that a product should not be simpler than its reactants [62]. The retrosynthetic accessibility score trains a classifier on the outcomes of an automated retrosynthetic planner, so that a molecule is scored by whether a route is likely to be found for it [63]. All three are cheap and easy to attach to a generative objective, and their limits should be stated with equal clarity: a critical assessment has shown that such scores function largely as fast surrogates for the behaviour of one particular planner rather than as general measures of synthesizability, and that they can be optimised against without any corresponding gain in real tractability [64].

The second family plans the synthesis explicitly rather than scoring a proxy for it. Computer-aided synthesis planning has moved from rule-based expert systems to neural retrosynthesis: Monte Carlo tree search combined with neural networks that propose transformations, filter them for feasibility and estimate the value of a position produces routes that chemists judge comparable to literature syntheses, and finds them far faster than earlier approaches [65]. Open implementations such as AiZynthFinder have made this routine and scriptable [66], so that a planner can be run directly as a filter on generated molecules at a per-molecule cost that is no longer prohibitive.

The third family changes the problem rather than mitigating it, by constraining generation so that only synthesizable molecules can be produced at all. Instead of generating a graph and asking afterwards whether it can be made, these methods generate a synthetic route and read the molecule off its end: the model’s action space is the set of available building blocks and reliable reactions, so that every sample arrives with a proposed synthesis attached. Amortised synthesis-tree generation established the approach [67], and reaction-based generative flow networks have extended it to the diverse, multi-objective sampling described in Section 2.4, reporting order-of-magnitude reductions in the estimated cost of making the molecules proposed [68]. A closely related and thoroughly industrialised expression of the same logic is the make-on-demand chemical space: enumerations such as Enamine REAL and ZINC-22 are defined by validated reactions applied to stocked building blocks, so that a compound chosen in silico can be delivered physically with a high success rate [69], and synthon-based screening navigates those spaces without enumerating them, as Section 4.6 describes.

The practical recommendation that follows is a layering of the same shape as the one that governs affinity prediction: constrain the generator to synthesisable space wherever the target permits it; score with a fast surrogate to rank within that space; and commit to an explicit retrosynthetic plan only to the small number of molecules that will actually be ordered or made. What none of this removes is the final step of a chemist’s judgement and a laboratory’s attempt, and the honest position is that the synthesizability gap has been substantially narrowed rather than closed. Its residual consequences are taken up in Section 9.

## 3. Protein Structure Prediction and the Expansion of the Druggable Proteome

### 3.1. The Structure-Prediction Revolution

Structure-based design of a molecule against a target presupposes a structure, and for much of the undruggable proteome experimental structures were scarce, partial, or captured in conformations irrelevant to drugging. The deep-learning prediction of protein structure has removed this bottleneck for an enormous fraction of proteins. AlphaFold2 achieved accuracy competitive with experiment at the level of individual domains, attaining median backbone errors below one angstrom for its highest-confidence predictions and providing, through the associated database, models for nearly the entire human proteome [70,71]. RoseTTAFold reached comparable accuracy through a three-track architecture coupling sequence, distance and coordinate information, and protein language models such as ESMFold subsequently demonstrated that structure can be predicted directly from a single sequence at scale, trading some accuracy for speed across hundreds of millions of proteins [72,73]. For the medicinal chemist confronting an undruggable target, the immediate consequence was the availability of a structural starting point where previously there had been none, and the ability to model mutants, paralogues and domain architectures rapidly.

### 3.2. Modelling Complexes and Ligands: AlphaFold3 and RoseTTAFold All-Atom

Predicting an isolated protein fold is necessary but not sufficient for drug design, which requires the structure of the protein in complex with ligands, ions, nucleic acids and partner proteins. The most consequential recent development is the extension of prediction to these biomolecular complexes. AlphaFold3 replaced the geometry-specific structure module of its predecessor with a diffusion module that operates directly on raw atomic coordinates, conditioned on a representation of the molecular system, and reported substantially higher accuracy than classical docking on protein–ligand complexes in the PoseBusters benchmark set [14]. RoseTTAFold All-Atom generalised the three-track approach to arbitrary small molecules, covalent modifications and assemblies, enabling joint modelling of proteins with their bound ligands within a single framework [15]. Together, these systems begin to make in silico the structure of a target-ligand complex the central object of structure-based design routinely accessible.

These capabilities carry important caveats that bear directly on their use for drugging hard targets. The ligand geometries produced by diffusion-based predictors can contain stereochemical errors incorrect chirality, distorted bond lengths and angles because the models optimise atomic positions without explicitly enforcing chemical constraints, prompting the development of restraint-guided inference that injects geometric priors during the reverse-diffusion sampling [74]. More broadly, as the PoseBusters analysis emphasised for docking generally, a predicted complex that scores well on a coordinate metric may nonetheless be physically invalid or may fail to generalise to targets unlike those seen in training, so predicted complexes must be treated as hypotheses to be validated by physics and experiment rather than as ground truth [53]. The point is not that the methods are unreliable but that their outputs occupy the same epistemic status as any other model: provisional, and most powerful when embedded in a pipeline that subjects them to rigorous downstream evaluation.

### 3.3. Cryptic and Allosteric Pockets: Drugging What Is Not Visible at Rest

The deepest reason many oncogenic proteins were deemed undruggable is that their ground-state structures display no suitable pocket. Yet proteins are dynamic, and a site absent in a static crystal structure may open transiently as the protein breathes among its accessible conformations. Such cryptic pockets, together with allosteric sites distinct from the orthosteric centre, are the key to the undruggable class, and their systematic discovery is one of the most important contributions of computation to the field [8]. Molecular dynamics simulation, which integrates Newton’s equations of motion for an all-atom system to sample conformational space, can reveal these transient openings, and enhanced-sampling methods are often required because the relevant motions are rare on simulation timescales [75,76]. Metadynamics accelerates sampling by depositing a history-dependent biasing potential that discourages the system from revisiting explored states, while replica-exchange molecular dynamics runs many copies at different temperatures and periodically swaps them to surmount energy barriers [77,78]. These techniques have repeatedly exposed cryptic pockets that became the basis for drug discovery campaigns against otherwise featureless surfaces [79].

Deep learning has now made cryptic-pocket discovery fast and predictive. PocketMiner, a graph neural network built on geometric-vector-perceptron layers and trained on a large corpus of molecular-dynamics data, predicts the locations of cryptic pockets directly from a single static structure, achieving an area under the receiver-operating-characteristic curve of 0.87 and running orders of magnitude faster than simulation; strikingly, its analysis suggested that more than half of proteins lacking an obvious ground-state pocket may nonetheless harbour a cryptic one [7]. The implication for oncology is profound: targets dismissed on the basis of a featureless crystal structure may in fact be druggable through sites that open only dynamically, and those sites can now be sought computationally before any experimental effort is committed. The discovery of the KRAS switch-II pocket, discussed in Section 5, is the proof of principle that a cryptic, induced site can convert an archetypally undruggable protein into a clinically tractable one.

### 3.4. Variant-Effect Prediction and Target Assessment

A final contribution of deep learning to the assessment of hard targets is the prediction of the functional consequences of sequence variation. AlphaMissense, built on the structural representations learned for folding, predicts the effect and pathogenicity of missense variants across the proteome, classifying a large fraction of all possible human missense changes as likely benign or likely pathogenic [80]. Its scope should be stated precisely, because it is easily overstated. It is a general, proteome-wide variant-effect predictor, trained without reliance on clinical labels and validated against clinical variant databases and multiplexed assays of variant effect; it is not a classifier of cancer drivers against passengers, it is not disease-specific, and its authors advance no such claim. What it supplies for oncology is a functional prior: a cheap, proteome-wide prioritisation of which sequence changes are likely to matter at all. The questions that actually govern a drug-discovery campaign whether a given somatic mutation drives a particular tumour, in which lineage, and whether it is therapeutically actionable require cancer-specific genomic, biological and clinical evidence that no general variant-effect model supplies. Used with that qualification, such predictions help narrow which mutant forms of a target merit dedicated attention the mutation-specific reactivation of p53-Y220C being a case in which the precise variant defines both the structural opportunity and the therapeutic strategy. Structure prediction thus contributes not only the coordinates on which design operates but also part of the rationale for choosing the target in the first place.

## 4. The Physics and Biochemistry of Binding

As in Section 2, the formalism below is preceded by the question it exists to answer. Generative design produces candidate molecules faster than any laboratory can test them, so the binding decision has to be taken computationally before it is taken experimentally, and the whole enterprise rests on how accurately that can be done. The quantity that matters is the binding free energy, because it, and not any geometric or learned score, determines occupancy at a given concentration. The question is how to compute it, at what cost, and with what error bar.

Three answers are developed below, and they differ by roughly three orders of magnitude in both cost and rigour. End-point methods such as MM-PBSA are cheap and approximate. Alchemical free-energy methods, of which the Zwanzig perturbation identity is the foundation, are expensive and reach roughly one kilocalorie per mole in favourable cases which is the accuracy at which a medicinal chemist’s decisions actually change; both are treated in Section 4.2. Molecular dynamics, in Section 4.3, supplies the conformational ensemble on which both depend, and is indispensable precisely for the cryptic pockets that define the undruggable class. Section 4.4 and Section 4.5 then treat two cases in which equilibrium affinity is the wrong quantity altogether: covalent inhibition, where the relevant measure is a rate rather than a constant, and residence time, where what matters is how long the complex survives in a body that is clearing the drug. Section 4.6 closes by setting out what each of these methods costs in practice, because a filter that cannot be afforded is not a filter.

### 4.1. The Thermodynamics of Molecular Recognition

Whether a designed molecule engages its target is, at equilibrium, a question of free energy. For the association of a protein P and ligand L to form a complex PL, the dissociation constant and the standard binding free energy are related by

*K_d = [P][L]/[PL], ΔG° = −RT ln K_a = RT ln K_d*

where K_a = 1/K_d, R is the gas constant, and T is the absolute temperature; a nanomolar K_d corresponds to a binding free energy near −12 kcal/mol at physiological temperature. The binding free energy decomposes into enthalpic and entropic contributions,

*ΔG = ΔH − TΔS*

and this decomposition is not academic for undruggable targets. Engaging a flat protein–protein interface or a shallow cryptic pocket often yields little enthalpic gain from hydrogen bonding and depends instead on hydrophobic burial and the entropy of displaced water, while binding a disordered region exacts an entropic penalty as the ensemble is restricted. The competition that made RAS intractable is likewise thermodynamic: any active-site ligand must achieve a binding free energy competitive with that of GTP present at millimolar concentration, a bar so high that the field abandoned the orthosteric site entirely in favour of allosteric pockets [3]. Quantifying binding free energy from structure is therefore the central predictive problem of structure-based design, and the methods that address it span a spectrum of rigour and cost.

### 4.2. Free-Energy Methods: From Perturbation Theory to Practical Accuracy

The most rigorous tractable route to relative binding free energies is alchemical free-energy calculation, grounded in statistical mechanics. The foundational result is the Zwanzig free-energy perturbation identity, which expresses the free-energy difference between two states A and B as an ensemble average over configurations sampled in state A,

*ΔG_{A→B} = −k_B T · ln*
 *⟨exp(−(U_B − U_A)/k_B T)⟩_A*

where U_A and U_B are the potential energies under the two Hamiltonians and the average is taken over the equilibrium ensemble of A [81]. In practice, the transformation between two ligands is carried out through a series of intermediate, non-physical states parameterised by a coupling variable λ, and the total free energy is obtained either by summing perturbation windows or by thermodynamic integration,

*ΔG = ∫_0^1*
 *⟨∂U(λ)/∂λ⟩_λ dλ*

To obtain the experimentally meaningful quantity the difference in binding affinity between two ligands one exploits a thermodynamic cycle, computing the alchemical transformation both in the bound complex and in solvent and taking the difference,

*ΔΔG_bind = ΔG_complex^{A→B} − ΔG_solvent^{A→B}*

so that the difficult absolute binding free energies cancel. A landmark prospective study established that a modern protocol combining accurate force fields with sufficient sampling reaches roughly one kilocalorie per mole accuracy across diverse targets and congeneric ligand series, sufficient to influence real medicinal-chemistry decisions [10]. A benchmark involving the harder case of ligands differing in net charge reported a root-mean-square error near 1.1 kcal/mol [82], and the most comprehensive public assessment to date, spanning more than a thousand compounds across twenty targets, both confirmed this performance and clarified its ceiling, estimating that the reproducibility of the underlying experimental affinities itself imposes a floor near 0.9 kcal/mol [83]. The candour of the field about the limits of these methods is essential: accuracy degrades for transformations involving large scaffold changes, net-charge changes, slow conformational rearrangements or significant protein reorganisation, and even an ideal calculation is bounded by the quality of the force field and of the calibrating data [84,85]. Free-energy perturbation is moreover computationally expensive, which positions it as a precise but costly filter to be applied to the most promising candidates rather than to an entire generated library. A cheaper, intermediate-accuracy alternative is the molecular-mechanics Poisson–Boltzmann (or generalised-Born) surface-area method, which estimates binding free energy from an end-point decomposition into molecular-mechanics energy, solvation and entropy terms,

*ΔG_bind ≈* *⟨E_MM⟩* *+* *⟨G_solv⟩* *− T⟨S⟩*

trading rigour for throughput and occupying a useful tier between docking and full free-energy perturbation [86]. The practical lesson, which recurs throughout structure-based design against hard targets, is that physics can rank candidates within a known and quantifiable margin of error, and that a generative pipeline should be engineered around that margin rather than in denial of it.

### 4.3. Molecular Dynamics and the Conformational Ensemble

Underlying both pocket discovery and free-energy calculation is molecular dynamics, the numerical integration of the classical equations of motion for an all-atom model of a biomolecular system in explicit solvent, using empirically parameterised force fields for bonded and non-bonded interactions [75]. Molecular dynamics yields not a single structure but a trajectory through conformational space, and it is this ensemble view that is indispensable for the undruggable class: cryptic pockets appear and disappear along the trajectory, allosteric couplings manifest as correlated motions, and intrinsically disordered regions are represented as distributions rather than fixed coordinates [76]. Because biologically relevant transitions are often slow relative to accessible simulation times, the enhanced-sampling methods already noted metadynamics, replica exchange and related approaches are frequently required to observe pocket opening or conformational interconversion within feasible computation [77,78]. The conceptual upshot is that the target of a structure-based campaign against a hard protein is often not a structure at all, but an ensemble, and the methods of molecular dynamics provide the means to characterise it.

### 4.4. The Kinetics of Covalent Inhibition

For several undruggable targets, the decisive strategy has been covalent inhibition, in which the ligand forms a chemical bond with a nucleophilic residue, and the relevant theory is kinetic rather than purely thermodynamic. Covalent inhibition typically proceeds in two steps: a reversible encounter complex forms, governed by an inhibition constant K_I, after which the covalent bond is formed at a rate k_inact,

*E + I*
 *⇌*
 *(E·I)*
 *→*
 *E*
 *−*
 I* [K_I, then k_inact]

The observed rate of inactivation as a function of inhibitor concentration follows a saturating relationship, and the single parameter that best captures the potency of an irreversible inhibitor is the second-order rate constant k_inact/K_I,

*k_obs = k_inact · [I]/(K_I + [I]), efficiency ≡ k_inact/K_I*

so that potency is not a single equilibrium affinity but a composite of recognition and reactivity; in the limit [I] ≪ K_I, the rate of target engagement is approximately (k_inact/K_I)[I], and time-dependent inhibition means that nominal IC50 values must be interpreted with the assay timeframe in mind [87]. This framework underlies the covalent KRAS-G12C inhibitors, which exploit the cysteine introduced by the G12C mutation, and it explains why such drugs can achieve durable, near-irreversible target occupancy that a reversible competitor could not [88]. The resurgence of covalent drugs across oncology, and the advances in tuning warhead reactivity to balance potency against off-target liabilities, reflect a maturation of exactly this kinetic understanding [89].

### 4.5. Drug-Target Residence Time and the Physiology of Exposure

A binding constant describes equilibrium, but cellular and clinical efficacy often track the lifetime of the drug-target complex, quantified by the residence time τ = 1/k_off, the reciprocal of the dissociation rate constant [90]. A long residence time can sustain target engagement even as free drug is cleared, decoupling pharmacodynamics from plasma pharmacokinetics, and is a design objective in its own right for targets where transient inhibition is insufficient. Connecting molecular potency to physiological effect requires the further apparatus of pharmacokinetic-pharmacodynamic modelling. The relationship between drug concentration C and biological effect is commonly captured by a sigmoidal Hill, or Emax, function,

*E = E_0 + (E_max · C^n)/(EC_{50}^n + C^n)*

in which E_max is the maximal effect, EC50 the concentration producing half-maximal effect, and n the Hill coefficient describing the steepness of the response; the same mathematical form as the Michaelis–Menten equation, v = V_max[S]/(K_M + [S]), governs the enzymatic turnover that many of these targets catalyse. For a small molecule to act on a tumour, it must in addition be absorbed, distributed, and able to penetrate tumour tissue at concentrations exceeding its EC50 for a sufficient duration, while sparing normal tissue enough to define a therapeutic window constraints summarised under the heading of ADMET. For covalent inhibitors, the in vivo extent of target engagement is governed not by a steady-state concentration but by the integral of exposure weighted by reactivity, so that the product of the unbound area under the concentration-time curve and the efficiency constant k_inact/K_I becomes the controlling quantity [87]. These physiological and pharmacological considerations are not peripheral: a molecule perfectly optimised against a structure in silico is therapeutically inert if it cannot reach its target in vivo, and the integration of design with pharmacology is part of what distinguishes a candidate drug from a computational artefact.

### 4.6. The Computational Economics of the Pipeline

The methods set out above differ not only in accuracy but in cost, by margins large enough to determine how a campaign is designed; anyone assessing such a campaign is entitled to know what it consumed. We therefore collect the published figures here, with the caveat that hardware, implementation and system size make any single number indicative rather than definitive, and that costs quoted in currency assume commodity cloud pricing at the time of the source publication. Table 5 summarises them.

At the cheap end, docking a single compound costs of the order of a CPU-second, and the economics of ultra-large screening follow directly. The campaign that established the approach docked 138 million make-on-demand molecules against a single target for approximately forty-four thousand CPU core-hours [11]; the VirtualFlow platform subsequently prepared a library of more than 1.4 billion commercially available compounds and screened over a billion of them against KEAP1, at a scale requiring institutional high-performance computing [91]. Because that cost grows linearly with library size while the libraries themselves have been growing faster than linearly ZINC-22 catalogues billions of tangible compounds, and the make-on-demand spaces behind it are an order of magnitude larger again [69] brute force has ceased to be the right strategy, and two responses have emerged. Synthon-based screening docks fragments and their combinations rather than enumerated products, and has searched a space of more than eleven billion compounds while explicitly docking under a tenth of a per cent of it [92]. Machine-learned triage trains a surrogate on a docked subset and uses it to filter the remainder, with reported accelerations of up to a hundredfold [93]. Both convert an intractable cost into a manageable one, and both introduce a surrogate whose own error must then be carried through the analysis [94].

At the expensive end, alchemical free-energy calculation costs are of the order of single-digit to tens of GPU-hours per ligand transformation, and that figure governs how the method can be used at all. A published analysis of active learning applied to a congeneric series of ten thousand compounds is the clearest illustration: running explicit free-energy calculations on roughly six per cent of the series recovered about three-quarters of the hundred best compounds, reducing the computation from the order of eighty thousand GPU-hours to under five thousand, which at the commodity rates assumed in that analysis is the difference between roughly one hundred and sixty thousand dollars and under ten thousand [95]. The same logic underlies reaction-based enumeration campaigns that explore hundreds of thousands of virtual ideas while committing full calculations to only a few thousand of them [96], and prospective deployment across many concurrent industrial projects has been documented at a scale of thousands of calculations [97]. The implication for pipeline design is unambiguous: free-energy perturbation is a decision instrument, to be spent on the compounds whose ranking will change a decision, and not a screen.

**Table 5:** Indicative computational cost and throughput of the principal methods used in a structure-based design campaign. Figures are as reported in the cited sources and depend on hardware, implementation and system size; they are intended to establish relative magnitude rather than to serve as benchmarks.

Method	Unit of Work	Reported Cost or Throughput	Source	Role in the Pipeline
Molecular docking, rigid receptor	One compound	Of the order of a CPU-second. A campaign over 138 million make-on-demand molecules against a single target consumed approximately 4.4 × 10^4^ CPU core-hours.	[11]	Primary triage.
Ultra-large virtual screening	10^9^ compounds	A library of more than 1.4 × 10^9^ compounds prepared and more than 10^9^ screened against a single target; requires institutional high-performance computing.	[91]	Exhaustive enumeration of tangible space.
Synthon-based screening	10^10^ compound space	A space of more than 1.1 × 10^10^ compounds searched while explicitly docking less than 0.1 per cent of it.	[92]	Navigating spaces too large to enumerate.
Machine-learned docking triage	Library scale	Up to one hundredfold acceleration relative to docking the full library, by training a surrogate on a docked subset.	[93]	Surrogate triage; introduces surrogate error.
MM-PBSA/MM-GBSA	One complex	Minutes to hours per complex, at intermediate accuracy.	[86]	Middle tier between docking and free energy.
Relative binding free energy (FEP/TI)	One transformation	Single-digit to tens of GPU-hours per perturbation edge; roughly 1 kcal/mol in favourable congeneric regimes.	[10,83,97]	Decision instrument, not a screen.
FEP under active learning	10^4^-compound series	Explicit calculation on approximately 6 per cent of a series recovered about 75 per cent of its best 100 compounds, reducing roughly 8 × 10^4^ GPU-hours to under 5 × 10^3^.	[95,96]	What makes free-energy methods affordable at series scale.
Generative model training, 3D diffusion	One model	Days on a small number of accelerators; reported inconsistently in the primary literature.	[12,13]	One-off cost, amortised over a campaign.
Generative sampling	One molecule	Negligible relative to the cost of evaluating the molecule produced.	[12,13]	Not the binding constraint on a campaign.

Generative model training sits between these poles and is the least well documented of the three, because training cost is reported inconsistently in the primary literature; structure-conditioned diffusion models of the kind described in Section 2.3 are typically trained on a small number of accelerators over days, and sampling from a trained model is cheap relative to evaluating what it produces [12,13]. The binding cost of a generative campaign is therefore almost never the generation itself but the physics and the chemistry downstream of it. Two conclusions follow, and both recur in Section 9. The first is that the pipeline’s cost is dominated by its accurate stages, so that the central design problem is one of triage: how to spend cheap, inaccurate computation in order to decide where to spend expensive, accurate computation. The second is that cost is not independent of the target. Cryptic-pocket and induced-fit systems require the enhanced sampling and ensemble treatment of Section 4.3, which can raise the cost of a single free-energy estimate by an order of magnitude and it is precisely the undruggable targets that fall into that class.

## 5. The Undruggable Targets: Biology and the Campaigns That Opened Them

The preceding sections have assembled a toolkit equivariant generative models, structure prediction, cryptic-pocket detection, and rigorous binding physics in the abstract. Its significance becomes concrete only when set against the specific biology of the proteins it is meant to defeat. This section therefore takes the four targets that have come to define the undruggable problem in oncology, and for each examines two things in turn: the molecular and cellular biology that made it intractable, and the campaign, increasingly computational, that has begun to overcome that intractability. The four the RAS GTPases, the tumour suppressor p53, the transcription-factor MYC, and the phosphatase SHP2 are chosen not for convenience but because each embodies a distinct mechanism of undruggability, and because each has yielded, partially or wholly, to a distinct combination of the methods set out above. Throughout this section we keep the three categories of Section 1.3 apart, because the history and the promise are not the same thing: what opened each target was, in every case, experimental and structural work supported by classical computation, and it is precisely by being clear about that record that the prospective claims for generative design can be assessed on their merits.

Table 1 sets out that mapping in summary form before the detailed treatment begins: for each target, the biological basis of its undruggability, the reason classical medicinal chemistry failed against it, the computational contribution classified by the three categories of Section 1.3, and the chemical matter and clinical status that resulted. The subsections that follow expand each row in turn.

### 5.1. KRAS: The Archetypal Undruggable Oncogene

#### 5.1.1. The GTPase Switch and Its Effector Network

RAS proteins are small guanine-nucleotide-binding proteins that function as binary molecular switches at the apex of the signalling networks controlling cellular proliferation, differentiation and survival. The switch is the nucleotide: in its GTP-bound state, RAS adopts a conformation, defined by the positions of two flexible loops termed switch I and switch II, that is recognised by downstream effectors; hydrolysis of the γ-phosphate to GDP relaxes these loops into an inactive conformation that effectors no longer bind. The cycle is regulated in trans by guanine-nucleotide exchange factors, which catalyse the release of GDP and thereby promote the active state, and by GTPase-activating proteins, which accelerate the otherwise slow intrinsic hydrolysis and thereby terminate the signal. Oncogenic mutations, overwhelmingly at glycine-12, glycine-13 or glutamine-61, act by impairing GTP hydrolysis most directly by rendering RAS insensitive to GTPase-activating proteins so that the protein accumulates in its active, effector-bound state and signals constitutively [3].

The active state engages a network of effectors of which two pathways dominate the oncogenic phenotype. The RAF–MEK–ERK mitogen-activated protein kinase cascade transmits a proliferative signal to the nucleus, while phosphoinositide 3-kinase signalling through AKT and mTOR governs growth and survival; both are engaged through direct binding to the switch regions of GTP-loaded RAS. KRAS specifically is the most frequently mutated oncogene in human cancer, with a mutational burden concentrated in three of the most lethal malignancies pancreatic ductal adenocarcinoma, colorectal carcinoma and non-small-cell lung cancer a distribution that made the protein simultaneously the most validated and the most coveted target in oncology, and for forty years the most conspicuous failure of small-molecule drug discovery.

#### 5.1.2. Why KRAS Resisted, and the Thermodynamic Argument

The reasons for that failure were specific and instructive. The first was thermodynamic. RAS binds guanine nucleotides with picomolar-to-femtomolar affinity, and the intracellular concentration of GTP is in the high-micromolar-to-millimolar range; a competitive, nucleotide-pocket-directed inhibitor would therefore need to achieve an affinity beyond anything realistic to displace the physiological ligand, leaving the orthosteric site effectively unavailable [3]. The magnitude of the problem can be stated compactly. If the fractional occupancy of the nucleotide site by an inhibitor I competing against GTP at concentration [G] follows the standard competitive expression,

*θ_I = [I]/([I] + K_I (1 + [G]/K_G))*

then with K_G in the picomolar range and [G] in the millimolar range, the term [G]/K_G reaches values of order 10^9^, so that meaningful occupancy by a competitive ligand would require K_I many orders of magnitude smaller still a demand no drug-like molecule can satisfy. The orthosteric route is, in effect, thermodynamically forbidden, and this single inequality explains why the obvious strategy was abandoned. The second reason was structural: the surface of RAS outside the nucleotide pocket is smooth and largely featureless, presenting no obvious cavity into which a small molecule might bind to perturb effector engagement allosterically. The protein appeared, on inspection of its crystal structures, to offer no foothold at all.

#### 5.1.3. The Switch-II Pocket: A Cryptic Site and Its Covalent Exploitation

**Figure 2:**
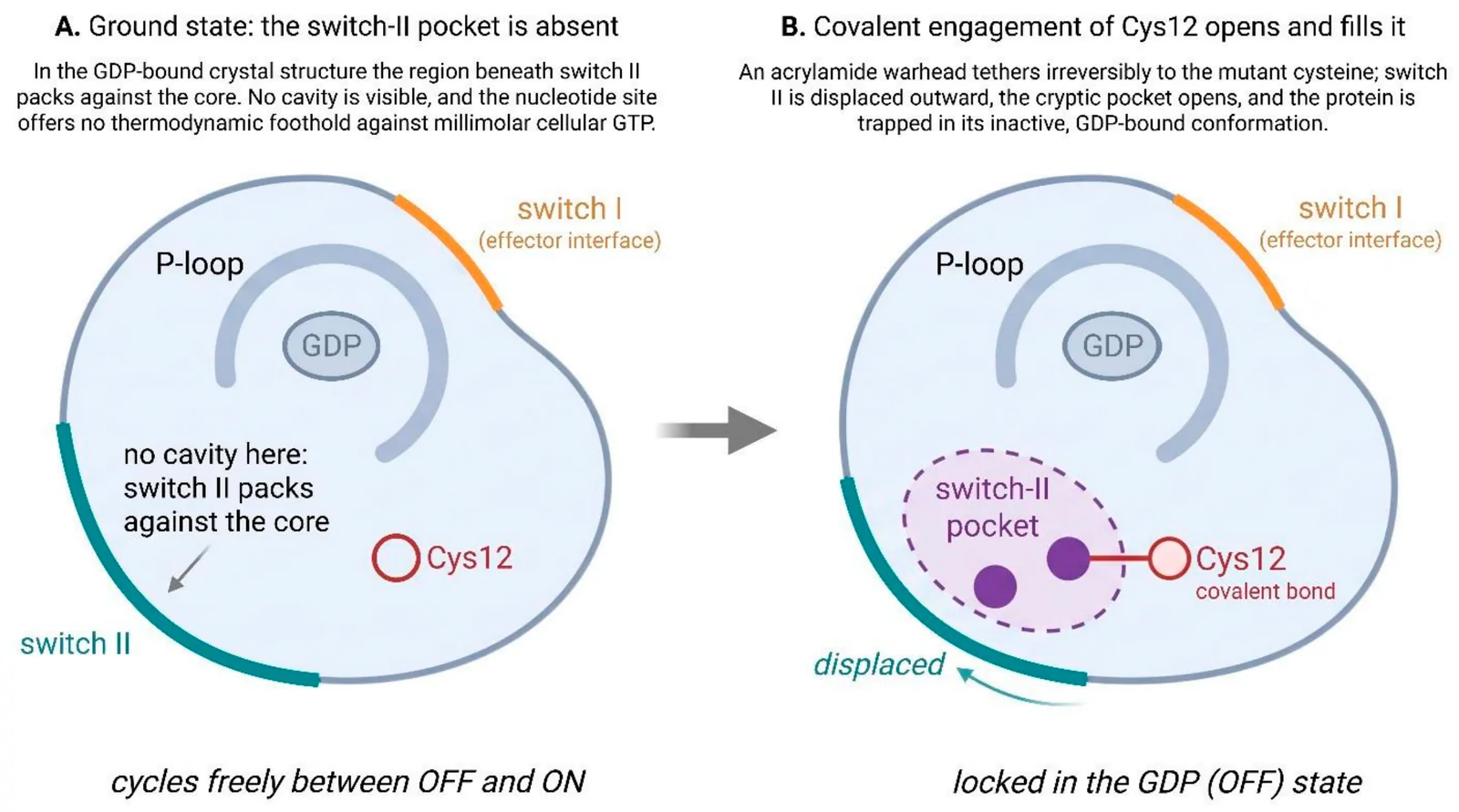
Schematic representation of the KRAS switch-II pocket, the cryptic site that converted the archetypal undruggable target into a clinically validated one. (**A**) In the ground state, the region beneath switch II packs against the core and no cavity is present; the nucleotide site offers no thermodynamic foothold against millimolar cellular GTP. (**B**) A covalent warhead tethers irreversibly to the mutant Cys12; switch II is displaced, the cryptic pocket opens, and the protein is trapped in its inactive, GDP-bound conformation with the effector interface disabled. Shapes are diagrammatic, are not drawn to scale, and do not represent experimental coordinates. Created in BioRender. Niazi, S. (2026) https://BioRender.com/zhy7hkf.

The impasse was broken not by raising affinity but by abandoning equilibrium competition altogether, in a strategy that is the single most important existence proof for the entire undruggable enterprise (Figure 2). In 2013, Ostrem, Shokat and colleagues reported that the cysteine introduced by the G12C mutation the most common KRAS mutation in lung adenocarcinoma could be exploited by small molecules bearing an electrophilic warhead that forms an irreversible covalent bond to that residue. Crucially, the resulting adducts bind not in the nucleotide pocket but in a previously unseen cleft beneath the switch-II loop, the switch-II pocket, which is essentially absent from the ground-state structure and opens only upon ligand engagement: a cryptic pocket in the precise sense developed in Section 3 [9]. Covalent attachment finesses the thermodynamic problem entirely, because an irreversible bond is not in competition with GTP; the kinetic framework of Section 4.4, in which potency is governed by the efficiency constant k_inact/K_I rather than an equilibrium affinity, is exactly the one that applies. The compounds preferentially engaged the GDP-bound state, and allele-specific inhibition of the mutant while sparing wild-type RAS proved achievable, addressing the selectivity concern that had shadowed the field [98].

Translation of this insight was rapid. Optimisation of the covalent switch-II strategy produced AMG510, sotorasib, the discovery of which was guided by structural and computational analysis of the pocket and which was the first KRAS inhibitor to enter the clinic and the first to be approved [99,100]; the same agent was subsequently approved in combination with panitumumab for KRAS-G12C-mutated metastatic colorectal cancer, extending the allele-specific strategy beyond lung cancer [101]. A second compound, MRTX849, adagrasib, followed from an independent campaign with the same covalent, switch-II-directed logic and an optimised pharmacokinetic profile [102]. Both demonstrated clinical activity in KRAS-G12C-mutant non-small-cell lung cancer in pivotal trials, converting the archetypal undruggable target into a source of approved medicines within a decade of the pocket’s discovery [103,104]. The lesson is general and is the thesis of this review in microcosm: the druggability of KRAS was not a fixed property of the protein but a function of the methods covalent chemistry, structural characterisation of a transient pocket, and kinetic rather than thermodynamic thinking brought to bear on it.

#### 5.1.4. Beyond G12C:G12D, Pan-RAS, and the Tri-Complex

The G12C success, however, addressed only a minority of KRAS-driven disease, because the G12C allele is common in lung cancer but rare in pancreatic and colorectal cancers, where other substitutions, above all G12D, predominate. G12D presents a harder problem: it lacks the reactive cysteine, so the covalent strategy is unavailable, and a non-covalent ligand must again contend with the thermodynamics of the switch-II pocket. The response was MRTX1133, a non-covalent, highly optimised inhibitor that occupies the induced switch-II pocket of KRAS-G12D with picomolar affinity, achieved through extensive structure-based design against a pocket that exists only when the ligand is bound [105]. That such affinity to a cryptic, non-covalent site is attainable at all is a striking result, and it reduces the conformational plasticity of the mutant protein well beyond the immediate vicinity of the pocket. Two qualifications belong with this example, because it is often cited loosely. The first concerns attribution: the published account describes crystallography-guided structure-based optimisation and does not report free-energy perturbation as a driver of that work, so MRTX1133 should be read as a validation of structure-based design against an induced pocket rather than as evidence for the alchemical methods of Section 4. The second concerns status: the clinical programme did not prosper, and the phase 1 study was terminated before the phase 2 portion opened [106], with the G12D effort passing to successor compounds. The sponsor has published no account of that decision; a company statement reported in the trade press indicated that no safety concern had been identified but that the pharmacokinetic data were highly variable and suboptimal [107], which, if accurate, locates the failure in the developability of the molecule rather than in the validity of the target. MRTX1133 therefore stands as an important chemical proof of principle rather than as an example of clinical translation, and Table 1 distinguishes it on that basis.

A conceptually distinct and more radical strategy abandons the inactive state entirely and targets active, GTP-bound RAS. RMC-6236, daraxonrasib, is an orally bioavailable molecule of beyond-rule-of-five dimensions that functions not as a simple inhibitor but as the organising component of a tri-complex: it binds the ubiquitous intracellular chaperone cyclophilin A, and the resulting binary complex presents a composite surface that engages the active, GTP-loaded state of RAS across a pocket formed jointly by cyclophilin A and the switch regions, sterically blocking effector binding [108]. Because the approach engages multiple RAS isoforms and mutant alleles in their active state, it is described as a pan-RAS(ON) inhibitor, and it has now completed that trajectory. In a randomised phase 3 trial in previously treated metastatic pancreatic adenocarcinoma, it met its dual primary endpoint in the RAS-G12 population, with a median overall survival of 13.2 against 6.6 months and a hazard ratio of 0.40, and returned a closely similar result in the broader enrolled population [24]; the FDA approved it in August 2026 for metastatic pancreatic adenocarcinoma in patients who have received at least one prior systemic therapy or who are not candidates for multiagent chemotherapy, an indication not restricted by RAS mutation status [109]. In an early-phase study at clinically relevant doses, the most common treatment-related adverse event was rash, occurring in the large majority of patients. The tri-complex strategy is significant beyond RAS as a demonstration that an intractable, flat protein surface can be rendered druggable by recruiting an endogenous protein to co-construct a binding site a logic continuous with the molecular glues of Section 6.

The same biology has been probed with non-small-molecule reagents that serve as both validation and chemical-biology tools. Synthetic monobodies, small engineered binding proteins, have been raised that recognise the switch-II pocket of KRAS-G12D selectively and non-covalently, mapping the conformational space of the pocket and confirming that high-affinity, mutant-selective engagement of this cryptic site is achievable in principle [110]. Such reagents illustrate how the boundary of druggability is explored: a pocket first revealed by one modality is characterised by another, and the accumulated structural knowledge feeds the computational models that design the next generation of small molecules.

### 5.2. p53: Restoring a Broken Tumour Suppressor

#### 5.2.1. The Guardian of the Genome and the Consequences of Its Loss

The tumour suppressor p53 is the most frequently mutated gene in human cancer and presents a problem categorically different from that of KRAS. Where KRAS is an oncogene activated by mutation, so that the therapeutic goal is inhibition, p53 is a tumour suppressor inactivated by mutation, so that the goal is restoration of a lost function a far less natural task for a small molecule, which can ordinarily block an activity more readily than it can recreate one. In its wild-type form, p53 is a transcription factor that, in response to genotoxic and oncogenic stress, activates programmes of cell-cycle arrest, senescence and apoptosis, thereby suppressing the propagation of damaged cells; its centrality to genome integrity earned it the description of guardian of the genome. The protein is held in check in unstressed cells principally by the E3 ubiquitin ligase MDM2, which binds the p53 transactivation domain, blocks its transcriptional activity and targets it for proteasomal degradation, in a feedback loop completed by p53-dependent transcription of MDM2 itself.

Two broad therapeutic situations follow from this biology. In tumours that retain wild-type p53 but suppress it through MDM2 overexpression, restoring p53 activity requires only that the p53–MDM2 interaction be disrupted; this is a protein–protein interaction of the kind discussed in general terms in Section 5.5, and it proved tractable. The Nutlins, identified by Vassilev and colleagues, are small molecules that occupy the p53-binding cleft of MDM2, displacing p53 and reactivating its tumour-suppressor programme, and they established the principle that a well-defined protein–protein interface can be drugged by a small molecule that mimics the key anchoring residues of one partner [111]. The harder and more common situation, however, is that of tumours bearing missense mutations in p53 itself, which both abolish its function and, frequently, exert dominant-negative and gain-of-function oncogenic effects of their own [112].

#### 5.2.2. The Y220C Mutant and a Druggable Crevice Created by Mutation

**Figure 3:**
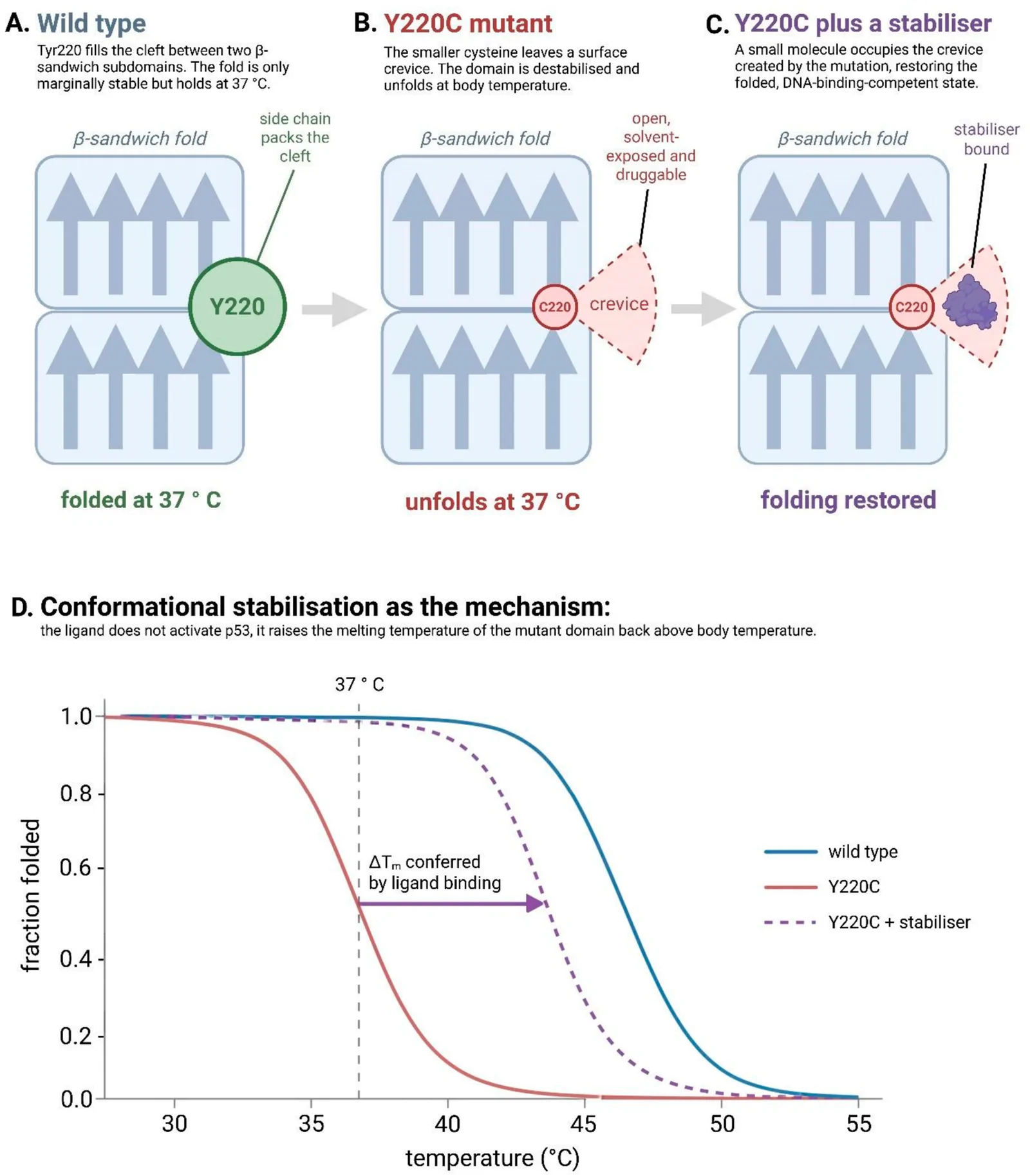
Schematic representation of the p53-Y220C crevice, a druggable cavity created rather than destroyed by an oncogenic mutation. (**A**) In the wild type, Tyr220 fills the cleft between two subdomains of the DNA-binding-domain β-sandwich. (**B**) Substitution by the smaller cysteine leaves a surface crevice and substantially destabilises the domain, reducing the population of the native-like folded state at near-physiological temperatures. (**C**) A small molecule occupying that crevice restores the folded, DNA-binding-competent state. (**D**) The mechanism is conformational stabilisation: the ligand does not activate p53 but increases the thermal stability of the mutant domain and shifts the equilibrium back toward the native-like conformation. The curves are illustrative and are not fitted to data; they are not intended to define a precise physiological unfolding threshold, and the transition should be read as a shift in a population equilibrium rather than as an abrupt switch. Created in BioRender. Niazi, S. (2026) https://BioRender.com/3mb8ifx.

The great majority of p53 mutations are missense substitutions clustered in the DNA-binding domain, and they fall into two mechanistic classes: contact mutations, which alter residues that touch DNA directly, and structural mutations, which destabilise the fold of the domain. The structural mutants are, paradoxically, the more approachable, because if the destabilising mutation can be compensated if the native fold can be pharmacologically stabilised then wild-type function may be restored. The exemplary case is the Y220C mutation. Substitution of the buried tyrosine-220 by cysteine removes a stabilising aromatic side chain and opens a surface crevice that does not exist in the wild-type protein, lowering the thermal stability of the domain so that it unfolds at physiological temperature; the mutation accounts for a small but significant share of all p53 mutations and is among the most frequent single substitutions [113]. The crevice it creates is, once again, a pocket born of mutation conceptually analogous to the cysteine of KRAS-G12C in that the very lesion that drives the cancer also furnishes the means of attacking it (Figure 3).

The therapeutic strategy is to design a small molecule that binds this mutation-induced crevice and, by filling it, restores the thermodynamic stability of the native fold, shifting the conformational equilibrium back toward the functional state a stabiliser rather than an inhibitor, exploiting exactly the ensemble and free-energy logic of Section 4. Structure-guided design against the Y220C pocket produced a series of stabilising ligands of increasing potency, culminating in clinical candidates [114]. The most advanced, rezatapopt (PC14586), is a first-in-class oral small molecule that binds the Y220C crevice, as confirmed by co-crystallography, and restores wild-type conformation and transcriptional function selectively in tumours bearing that mutation [115]. The clinical evidence should be read in its proper order and hierarchy. The peer-reviewed phase 1 study, conducted across tumour types in seventy-seven heavily pretreated patients with TP53-Y220C-mutant solid tumours, reported an objective response rate of twenty per cent overall, rising to thirty per cent among patients whose tumours were KRAS wild-type and who received at least 1150 mg once daily [115]. That is the pan-tumour proof of concept for the pharmacological reactivation of a mutant tumour suppressor, and a result that would have seemed scarcely conceivable a decade earlier. The ovarian experience is a separate and later body of evidence. A phase 1 subgroup analysis presented in 2024 described responses in roughly half of a fifteen-patient group, and the registrational phase 2 portion of the basket trial, whose protocol has been published [116], has since reported an interim analysis of its ovarian cohort in which the objective response rate was approximately forty-four per cent among seventy-two evaluable patients. The two bodies of evidence should not be conflated: the twenty per cent figure describes the published pan-tumour phase 1 population, while the higher figures describe a lineage-restricted cohort within a single-arm registrational trial whose primary results have not yet appeared in full. The response is not uniform across lineages: in Y220C-mutant myeloid disease the compound restored the native conformation but produced only limited apoptotic response, underscoring that conformational rescue is necessary but not always sufficient, and that the cellular context into which a restored p53 is delivered conditions the outcome.

### 5.3. MYC: Targeting an Intrinsically Disordered Master Regulator

If KRAS is the archetype of the thermodynamically defended target and p53 of the lost function to be restored, MYC is the archetype of the intrinsically disordered target, and it remains among the hardest of all. MYC is a transcription factor that, by binding throughout the genome, acts as a general amplifier of the transcriptional programme, and its deregulation is among the most common events in human cancer; it is causally implicated in a large fraction of malignancies, and its experimental inactivation causes regression of established tumours, so that its validity as a target is beyond question. The difficulty is structural in the most fundamental sense. The functional, DNA-binding form of MYC is a heterodimer with its partner protein MAX, assembled through a basic helix–loop–helix leucine zipper interface, and in the absence of MAX the relevant region of MYC is intrinsically disordered, populating an ensemble of interconverting conformations rather than a single fold [5]. There is, in the monomeric protein, no stable pocket to bind and no fixed structure against which to design; the very framework of structure-based drug discovery, which presumes a target structure, appears not to apply.

The biophysics of intrinsic disorder, set out in general in Section 3 and Section 4, here becomes the central obstacle. A disordered protein is correctly described not by coordinates but by a distribution over conformations, and a ligand that engages it must either select and stabilise a minor folded sub-state from that ensemble or bind in a manner that itself remains partly disordered a so-called fuzzy complex. Both possibilities lie at the edge of what current design methods can reliably achieve, and the conformational-ensemble methods of molecular dynamics are essential simply to characterise what is being targeted. For these reasons, direct small-molecule inhibition of MYC has not yet succeeded, and the most advanced approaches have been protein-based, serving meanwhile as validation that the MYC–MAX interaction can be disrupted therapeutically at all.

The leading such approach is Omomyc, a dominant-negative miniprotein derived from the MYC leucine-zipper that was originally devised as a genetic tool to validate MYC inhibition in vivo, where it caused dramatic tumour regression with tolerable and reversible side effects [117]. The translation of Omomyc from a genetically expressed construct into a therapeutic depended on the unexpected discovery that the purified protein possesses intrinsic cell-penetrating activity and can be administered as an agent in its own right, entering cells and disrupting MYC–MAX dimerisation and DNA binding from within [118]. Its clinical derivative, OMO-103, completed a first-in-human trial in patients with advanced solid tumours, establishing safety and yielding evidence of disease stabilisation together with pharmacodynamic confirmation of target engagement the first clinical validation of direct MYC targeting, and a demonstration that even this most disordered of targets is not categorically beyond reach [119]. It should be said plainly that Omomyc is an achievement of protein engineering and structural biology rather than of machine learning: no part of its design was generative, and it is cited here as evidence that the target is addressable, not as evidence of what artificial intelligence can do. The case also delineates the current frontier with unusual sharpness: the protein modality has reached the clinic while the small-molecule, and a fortiori the generatively designed small-molecule, approach to MYC remains an open problem, and one for which the ensemble-aware generative methods of Section 2 are the most plausible eventual route.

### 5.4. SHP2 and the Protein Tyrosine Phosphatases: Allostery as the Solution

The protein tyrosine phosphatases constitute a large enzyme family long regarded as undruggable for a reason different again from those above: the chemistry of their active site. The catalytic pocket of a phosphatase is built to bind a phosphotyrosine, and is accordingly highly polar and positively charged; ligands designed to occupy it competitively have tended to be correspondingly charged and polar, with poor membrane permeability and poor pharmacokinetics, and the high conservation of the active site across the family made selectivity between phosphatases extremely difficult [120]. For SHP2, the phosphatase encoded by PTPN11 and a central positive transducer of signalling from receptor tyrosine kinases into the RAS–ERK pathway, these generic difficulties were compounded by the protein’s importance: SHP2 sits upstream of RAS, and its inhibition offered a route to dampen oncogenic signalling even where RAS itself was the driver, making it a target of great interest and great frustration.

The resolution came from the protein’s own regulatory architecture. SHP2 is auto-inhibited: in its resting state, its two SH2 domains fold against the catalytic domain, occluding the active site, and activation requires that engagement of the SH2 domains by phosphorylated partners release this clamp and expose the catalytic site. This conformational equilibrium between closed and open states is precisely the kind of allosteric machinery that a small molecule can exploit without ever touching the polar active site. Chen, LaMarche and colleagues identified SHP099, a small molecule that binds a tunnel-shaped allosteric pocket at the interface of the three domains, present only in the closed conformation, and stabilises that auto-inhibited state, thereby blocking activation; its affinity was in the high-nanomolar range and, decisively, it was selective and cell-permeable in a way that no active-site inhibitor had been [121]. Mechanistically, the compound acts by binding and stabilising a particular conformation of the protein, a logic identical to the conformational stabilisation of p53-Y220C and, more broadly, to the molecular glues of Section 6, in that the ligand functions by shifting a conformational equilibrium rather than by occluding a catalytic site.

Optimisation of this allosteric series produced TNO155, a highly potent, selective and orally efficacious clinical candidate with low-nanomolar potency, which entered clinical trials and, importantly, is being developed in combination with the KRAS-G12C inhibitors discussed above [122]. The rationale for that combination connects the two targets in a single network argument: inhibition of mutant KRAS provokes adaptive reactivation of upstream receptor-tyrosine-kinase signalling that flows through SHP2, so that co-inhibition of SHP2 blunts a principal route of resistance. The phosphatase story thus illustrates two general themes at once that allostery is a general route into enzyme families whose active sites are undruggable, and that the undruggable targets are not isolated but embedded in signalling networks whose logic dictates rational combinations.

### 5.5. Transcription Factors, Protein–Protein Interactions, Fusions and Disorder

The four targets examined above are representatives, and it is worth stating explicitly the broader classes they stand for, since the same principles extend across them. Transcription factors as a class share the difficulty exemplified by MYC: they act through protein–protein and protein-DNA interfaces rather than catalytic sites, are frequently disordered in functionally important regions, and have accordingly been among the least tractable of all targets, even as they include many of the most important oncogenic drivers [4]. The strategies emerging against them stabilising or disrupting defined interaction interfaces, exploiting rare folded sub-states, and, increasingly, removing the protein entirely by induced degradation rather than inhibiting it recur throughout this review and are taken up mechanistically in Section 6.

Protein–protein interactions, more generally, were long considered undruggable because their interfaces are large, flat and lacking the concave pockets that bind small molecules; the success of the MDM2–p53 inhibitors and of fragment-based approaches to other interfaces has shown that a subset of such interfaces possess hot spots and transient pockets that can, in fact, be engaged, and the computational detection of exactly such cryptic sites, treated in Section 3, is what has systematised the search [6]. Oncogenic fusion proteins, generated by chromosomal rearrangement, present yet another variant: the fusion junction may create a neomorphic surface or constitutively activate a kinase or other enzyme, and while fusions involving druggable enzymatic domains have been addressed by conventional inhibitors, those that act through disordered or interaction-dependent mechanisms inherit all the difficulties of the disordered class. Across all of these, the unifying scientific content is that of intrinsic disorder and its biophysics: where there is no fixed structure, the target is an ensemble, classical pockets are absent or transient, and the methods that succeed are those ensemble characterisation by molecular dynamics, cryptic-pocket prediction, conformational stabilisation, induced proximity, and the generative design of molecules against all of these that this review has placed at its centre [5].

Two transcription-factor targets deserve to be named explicitly, because they are the ones most often paired with MYC in discussions of this class and because both were considered for detailed treatment here. STAT3 is activated by phosphorylation-dependent dimerisation through an SH2 domain, and although that domain is considerably more pocket-like than anything MYC offers, the campaign against it has repeatedly foundered on selectivity against other members of the STAT family and on the difficulty of achieving sufficient unbound exposure in the nucleus a variant of the paralogue-selectivity problem that Section 5.4 treats for the phosphatases. β-catenin, the transcriptional effector of WNT signalling, presents an armadillo-repeat superhelix that engages TCF and LEF factors across an interface of exactly the dimensions that make protein–protein interactions hard, with the additional difficulty that the same surface is used by physiological partners whose disruption carries intestinal toxicity. In both cases, the methods that apply are those developed here for MYC ensemble characterisation, interface hot-spot detection, and the substitution of induced degradation for occupancy which is precisely why they are treated by generalisation rather than separately.

It is equally worth being explicit about what does not belong in this category, since the boundary is often drawn too generously. The BCL-2 family, frequently cited as an undruggable success story, is better understood as the precedent that established that protein–protein interfaces contain druggable hot spots at all: the fragment-based, structure-guided campaign that produced navitoclax and then venetoclax was conducted largely before the generative era and did not depend on it. The epigenetic reader and writer domains bromodomains, methyltransferases, demethylases possess well-formed, conventionally druggable pockets, and their difficulties have been ones of selectivity, on-target toxicity and therapeutic index rather than of tractability; they were never undruggable in the sense used here. And the non-KRAS RAS isoforms are not a separate problem but the same one: NRAS and HRAS share the switch architecture, the nucleotide affinity and the effector interface of KRAS, differing chiefly in their hypervariable regions, their membrane anchoring and their mutational spectra across tumour types, so that the analysis of Section 5.1 transfers directly and the pan-RAS(ON) agents discussed there are designed precisely to exploit that shared architecture.

## 6. Modalities: Expanding What a Small Molecule Can Do

A recurring theme of the preceding section is that the undruggable targets have yielded not to better versions of conventional inhibitors but to fundamentally different modes of action. The covalent engagement of KRAS, the conformational stabilisation of p53 and SHP2, and the recruitment of a chaperone to build a binding site for active RAS each represent a departure from the classical paradigm of reversible, orthosteric, single-protein inhibition. This section treats these expanded modalities systematically, with the equilibrium and kinetic theory that govern them, because it is the enlargement of the medicinal chemist’s repertoire as much as any computational advance that has redrawn the boundary of druggability, and because each modality poses a distinct modelling problem for the artificial-intelligence methods that increasingly drive their design.

### 6.1. Covalent Inhibition

Covalent inhibition, whose kinetics were developed in Section 4.4, is the modality that broke KRAS, and its logic generalises. By forming a chemical bond to a nucleophilic residue most often a cysteine a covalent ligand escapes the equilibrium competition that defends targets with high-affinity natural ligands, and can achieve durable target occupancy governed by the efficiency constant k_inact/K_I rather than by a dissociation constant [87]. The strategy’s renaissance reflects a refined understanding of how to tune warhead reactivity: too reactive a warhead engages off-target nucleophiles and incurs toxicity and idiosyncratic liabilities, while too inert a warhead fails to label the target within a physiologically useful time, so that the design problem is the simultaneous optimisation of non-covalent recognition (which sets K_I and hence selectivity) and intrinsic reactivity (which sets k_inact) [88]. The availability of a uniquely placed mutant cysteine, as in KRAS-G12C, is the ideal case because it confers selectivity for the oncogenic protein at the level of covalent chemistry itself; the broader application of covalent strategies depends on identifying targetable nucleophiles near functionally important sites, a search to which structure prediction and pocket analysis directly contribute [89].

### 6.2. Allosteric Modulation

Allosteric modulation binding at a site distinct from the orthosteric or catalytic site to alter a protein’s activity through conformational change is the modality that solved SHP2 and that underlies the stabilisation of p53-Y220C, and it is perhaps the most general answer to undruggability. Its power rests on two facts. First, allosteric sites need not share the polar, conserved, ligand-defended character of orthosteric sites, so they are frequently more drug-like and more amenable to selective engagement across paralogous families, as the SHP2 case showed against the otherwise intractable phosphatases [121]. Second, because an allosteric ligand acts by shifting a conformational equilibrium, it can either inhibit or activate, and can stabilise specific states the closed, auto-inhibited SHP2, or the folded p53-Y220C that pure orthosteric occlusion could never select [98]. The cost is that allosteric sites are often cryptic, present only in a subset of conformations, which is precisely why their discovery has depended on the molecular-dynamics and machine-learning methods of Section 3; an allosteric pocket that opens only transiently will not be found in a single crystal structure but only in the ensemble.

### 6.3. Molecular Glues

The molecular glues represent a conceptual inversion of conventional inhibition: rather than blocking a function, a molecular glue induces a new protein–protein interaction, typically by binding at the interface between two proteins and stabilising a ternary complex that would not otherwise form. The therapeutically most developed application is induced degradation, in which a glue recruits a target protein to an E3 ubiquitin ligase, marking it for proteasomal destruction. The mechanism was first understood retrospectively for clinical agents whose mode of action had been mysterious: the anticancer aryl-sulfonamides, indisulam and its congeners, were found to act by recruiting the splicing factor RBM39 to the CRL4 ligase substrate-receptor DCAF15, inducing the polyubiquitination and degradation of RBM39 and thereby disrupting pre-messenger-RNA splicing [123,124]. Structural studies revealed how this works at the atomic level and, in doing so, exposed the central biophysical peculiarity of glues: the sulfonamide binds a shallow, non-conserved pocket on DCAF15 and neo-functionalises it as an interface for RBM39, with the ternary complex stabilised cooperatively even though the individual binary affinities are weak [125,126].

That cooperativity is the quantitative heart of the matter, and it is shared with the degraders of Section 6.4, so we set out its theory here. Consider a glue or degrader G that bridges a target T and a presenter or ligase E. The assembly of the ternary complex T·G·E can be described by the binary dissociation constants for the two pairwise interactions and by a dimensionless cooperativity factor α that measures how much the binding of one protein is enhanced (or impaired) by the prior binding of the other. Writing K_d for the binary target–glue interaction and K_d′ for its value in the presence of the ligase,

*α = K_d/K_d′ = K_{d,E}/K_{d,E}′*

so that α > 1 denotes positive cooperativity, in which the ternary complex is more stable than the binary affinities alone would predict, and α < 1 denotes negative cooperativity. Positive cooperativity is what allows molecular glues to function with binary affinities so weak as to be almost undetectable in isolation: the ternary complex is stabilised by direct, glue-induced protein–protein contacts between target and presenter, and it is this surface complementarity, rather than the intrinsic affinity of the glue for either protein, that determines success. Direct measurement of indisulam-family ternary complexes found apparent dissociation constants in the low-micromolar range with no detectable binary affinity in the absence of compound, the defining signature of a cooperative, glue-dependent interaction [126]. The rational design of molecular glues remains extraordinarily difficult precisely because it requires predicting and optimising an induced protein–protein interface rather than a single binding event, and the systematic discovery of glues has so far relied more on serendipity, phenotypic screening and high-throughput approaches such as DNA-encoded libraries than on de novo design [127]. It is for this reason that we identify generative, structure-based design of molecular glues against oncology targets as one of the principal open frontiers of the field: the modality is among the most powerful for undruggable proteins, yet the least served by current artificial-intelligence methods, whose ternary and induced-fit modelling capabilities remain immature.

**Figure 4:**
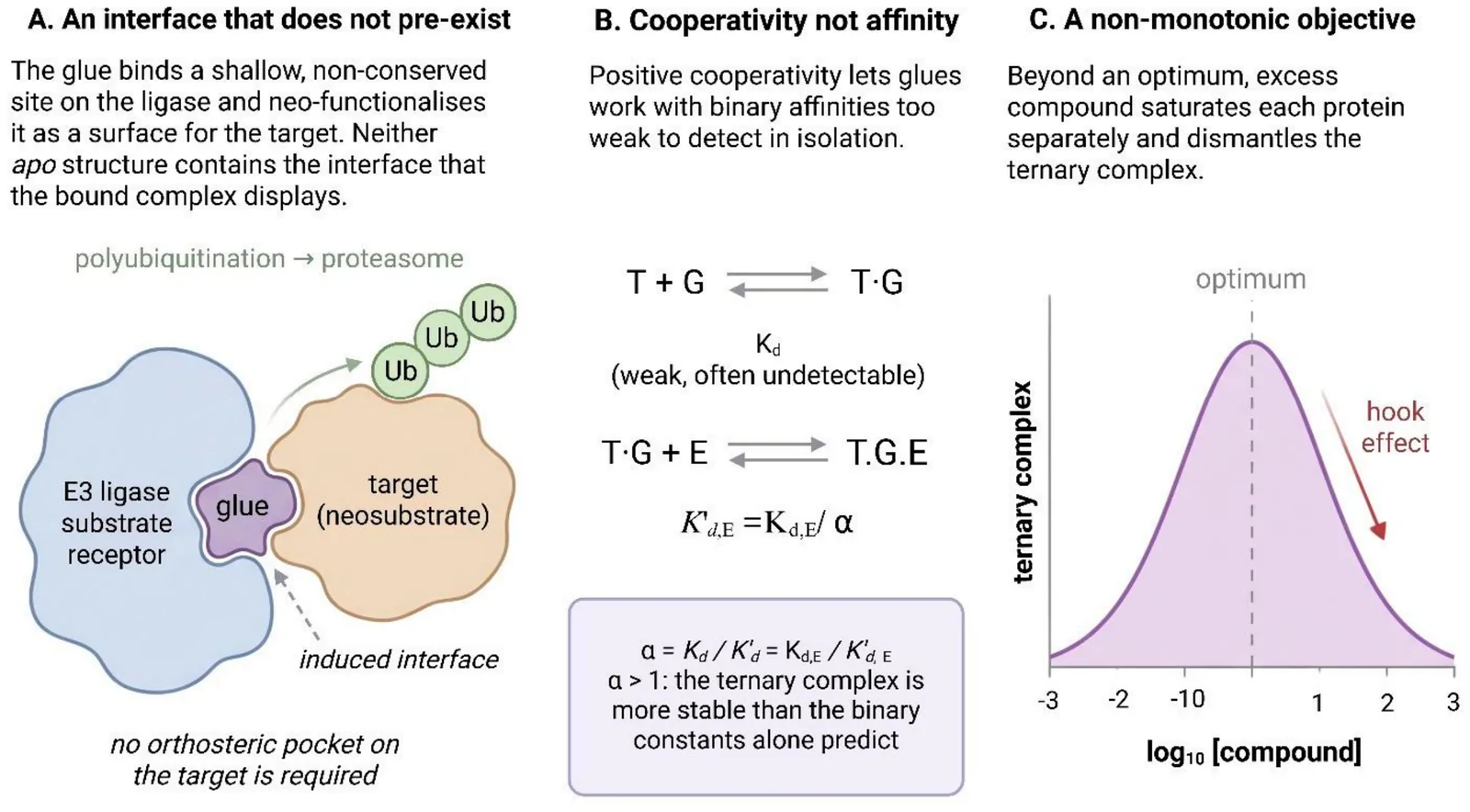
Ternary complex dynamics for a molecular glue, and why de novo design of this modality remains the hardest computational problem considered in this review. (**A**) The glue binds a shallow, non-conserved site on the ligase and neo-functionalises it as a surface for the target; neither apo structure contains the interface that the bound complex displays, so there is no pocket on which to condition a generative model. (**B**) Success is governed by cooperativity rather than by binary affinity, which is why glues can operate with binary constants too weak to measure in isolation. (**C**) Ternary complex formation is non-monotonic in compound concentration the hook effect so that an objective defined as maximum affinity is mis-specified. Diagrammatically, panel (**C**) plots the standard three-body equilibrium form rather than data for any particular compound. Created in BioRender. Niazi, S. (2026) https://BioRender.com/z30n55u.

That difficulty deserves a more specific diagnosis than the observation that it is hard, because the obstacles are distinct; they have different remedies, and identifying which of them binds in a given programme determines which method to reach for. Six are worth separating. Figure 4 illustrates the first, third and fourth, and Table 6 summarises all six together with the computational responses now emerging against each.

The first is that there is no pocket on which to condition. Every structure-based generative method described in Section 2 takes a receptor cavity as its input and designs a ligand to fill it. For a molecular glue, the relevant cavity on the target side does not exist before the compound arrives: the interface is created by the act of binding, and the shallow site on the presenter protein that the glue occupies is frequently not a recognisable pocket either. The conditioning signal that structure-based design requires is therefore simply absent, and the problem is not one of generating a ligand into a cavity but of generating the cavity itself.

The second is that the object to be modelled is a three-body complex in which two protein surfaces are free to reorganise. A binary docking problem has six rigid-body degrees of freedom plus ligand torsions; a ternary problem adds the full relative orientation of two proteins, each with its own induced fit, and the resulting search is not merely larger but qualitatively different in character. Independent benchmarking bears this out. An evaluation of current co-folding models against experimentally determined glue ternary structures released after the models’ training cut-off found that the best of them recovered only about a third of the glue–protein interactions and roughly half of the protein–protein interfaces [128]. A parallel assessment on PROTAC ternary complexes reported that apparent accuracy is substantially inflated by accessory subunits such as DDB1 and Elongin B/C, which contribute buried interface area without reflecting any degrader-specific recognition [129]. A comparison of AlphaFold3 with Boltz-2 across both modalities found performance strongly dependent on the ligase involved, with characteristic failures of global orientation [130]. The capability that ternary design would require has, in short, now been measured, and found wanting.

The third is that the quantity to be optimised is a cooperativity, and a cooperativity is a difference in free energies across a large, solvated protein–protein interface. Section 4.2 established that free-energy methods reach roughly one kilocalorie per mole for congeneric ligand series in well-behaved pockets; no comparable claim can be made for an induced protein–protein interface, where desolvation of broad polar surfaces and networks of ordered interfacial water dominate the energetics, and where the available scoring functions were parameterised on buried, drug-like cavities. The error bar on a computed α is therefore of the same order as the effect being designed for, which makes such calculations useful for ranking within a closely related series and unreliable as absolute predictions.

The fourth is that the biological objective is not monotonic in the quantity being computed. Degradation is a multi-step catalytic process assembly of the ternary complex, correct positioning of an accessible lysine relative to the E2 active site, processive ubiquitin transfer, proteasomal engagement, and release of the glue to act on the next target molecule and its efficiency is not a monotonic function of ternary affinity. Beyond an optimum, excess compound saturates the two proteins separately and dismantles the very complex it was designed to form, the hook effect shown in Figure 4C. A generative objective defined as maximising ternary affinity will therefore, past that point, actively degrade the property of interest; and no improvement in binding prediction repairs a mis-specified objective.

The fifth is data scarcity compounded by systematic bias. Experimentally determined glue-induced ternary complexes number in the low hundreds in total, and in the tens once restricted to structures released after model training, which is the only honest basis for evaluation [128]; and they are drawn overwhelmingly from a handful of ligases, principally cereblon and DCAF15 within the CRL4 family. Of the several hundred E3 ligases encoded in the human genome, only a small minority has been productively liganded at all. A model trained on that distribution has in effect learned the idiosyncrasies of two or three presenter proteins, which is why its performance degrades sharply when it is asked about a fourth, and why the discovery of glues has historically come from chemical-genetic and phenotypic routes rather than from design: correlating the cytotoxicity of compound collections with ligase expression across cancer cell lines identified cyclin K degraders whose mechanism no structure-based method would have proposed [131,132].

The sixth is that glue structure-activity relationships are unusually discontinuous. Because activity depends on complementarity across an induced interface rather than on occupancy of a defined cavity, minor chemical changes can create or abolish degradation outright, so that the smooth local landscapes which gradient-based and reinforcement-learning optimisers exploit are not reliably present one reason the diversity-seeking flow-network formulations of Section 2.4 are a better structural fit to this modality than single-objective optimisation.

The remedies now being explored map onto these obstacles individually rather than onto the modality as a whole, which is the practical value of separating them. Co-folding architectures are being adapted specifically to ternary prediction, and steered or guided sampling is being used to bias generation toward the induced interface; benchmark sets restricted to glue and degrader complexes have now appeared, which is the precondition for progress that can be measured rather than asserted [128,130]; and the chemical-genetic route retains the decisive advantage of finding glues whose mechanism nobody anticipated, which suggests that the productive near-term posture is a hybrid in which computation prioritises and rationalises rather than originates [20]. Our assessment therefore stands: this is the modality most valuable for undruggable targets and least served by current artificial intelligence. But it is now a set of specified problems rather than a single opaque one, and that is a meaningful change.

### 6.4. PROTACs and Targeted Protein Degradation

Where molecular glues are typically monovalent and exploit fortuitous surface complementarity, proteolysis-targeting chimaeras, or PROTACs, are designed bivalent molecules: a ligand for the target protein and a ligand for an E3 ligase, joined by a linker, so that the molecule recruits the ligase to the target deliberately and catalytically [133]. The approach, pioneered by Crews, Deshaies and colleagues, generalises induced degradation from the serendipitous case to a rational platform, and its development into cell-permeable, fully synthetic degraders recruiting the cereblon and von Hippel–Lindau ligases opened a vast target space, including proteins with no enzymatic activity to inhibit and no orthosteric pocket of consequence since a degrader need only bind the target somewhere sufficient to support ternary complex formation, not at a functionally critical site [134,135]. This event-driven, catalytic pharmacology, in which one degrader molecule can direct the destruction of many target molecules, is mechanistically distinct from occupancy-driven inhibition and is especially suited to the undruggable classes, where a tight functional pocket is exactly what is missing [136].

The governing physics is the ternary-complex equilibrium already introduced, and the cooperativity factor α is again decisive: structural and biophysical analysis of degrader ternary complexes has shown that productive degradation correlates with positive cooperativity arising from favourable target-ligase protein–protein contacts induced by the bound degrader, so that the linker and the two warheads must be co-optimised to present compatible surfaces [137]. A characteristic and practically important consequence of the bivalent mechanism is the hook effect: as degrader concentration increases beyond an optimum, the molecule increasingly saturates the target and the ligase in separate binary complexes rather than bridging them, so that the abundance of the productive ternary complex falls and degradation efficiency declines, giving a biphasic concentration-response. The phenomenon is a direct prediction of the coupled-equilibrium model the concentration of ternary complex is non-monotonic in total degrader and exemplifies why degrader pharmacology cannot be reduced to a single affinity and why its quantitative modelling, increasingly a target for machine-learning methods, must represent the full ternary system [138].

### 6.5. Polypharmacology and Network-Directed Design

A final modality is conceptual rather than chemical: the deliberate design of molecules to act on multiple targets, polypharmacology, in recognition that the robustness of oncogenic signalling networks often defeats the inhibition of any single node [139]. The undruggable targets are embedded in exactly such networks, and several themes already encountered the combination of SHP2 with KRAS inhibition to forestall adaptive resistance, the engagement of multiple RAS isoforms by a pan-RAS agent are expressions of a network rather than single-target logic. Designing for a defined multi-target profile, rather than for maximal selectivity against one protein, inverts a long-standing assumption of medicinal chemistry and poses a distinctive optimisation problem, one to which multi-objective generative models and the property-conditioned design methods of Section 2 are naturally suited, since they can in principle be conditioned on an activity profile across several targets simultaneously.

## 7. From in Silico to the Clinic: Existence Proofs and Translational Reality

### 7.1. What Has Actually Reached the Clinic

A field as laden with expectation as artificial-intelligence drug discovery must be assessed against what it has actually delivered, and here it is essential to distinguish demonstrations of principle from clinical outcomes, and successes in adjacent domains from successes against undruggable oncology targets specifically. The earliest widely cited demonstration was the use of a generative reinforcement-learning model to design inhibitors of the kinase DDR1, in which the system proposed novel compounds, a small number of which were synthesised and shown to be active in biochemical and cell-based assays, with a lead exhibiting favourable pharmacokinetics in mice [140]. The result was important as an early end-to-end demonstration that a generative model could yield real, active chemical matter, though it was also criticised for relying on substantial prior pharmacophore knowledge of the target and for excluding model-development time from its widely quoted timeline; it is best read as a proof that the generative-design loop can close, not as evidence of de novo discovery against a hard target.

A second and methodologically cleaner class of existence proof comes from antibiotic discovery, where a graph neural network trained to predict antibacterial activity, and then applied to screen large compound collections, identified halicin a molecule structurally remote from existing antibiotics with broad-spectrum activity confirmed in vivo and, in subsequent work, additional structurally novel antibacterial classes through models whose predictions were rendered interpretable [141,142]. These results matter for oncology not because the targets are shared but because they demonstrate, prospectively and with experimental confirmation, that a learned model trained on molecular structure can identify genuinely novel, functional chemical matter that human medicinal chemists had not found the core capability on which any application to undruggable targets depends.

The most consequential recent milestone is the first clinical proof of concept for a drug in which both the target and the molecule were products of generative artificial intelligence. Rentosertib, an inhibitor of the kinase TNIK a target itself nominated by a machine-learning analysis of disease biology was advanced into a randomised, placebo-controlled phase 2a trial in idiopathic pulmonary fibrosis, conducted in seventy-one patients over twelve weeks [143]. The design deserves to be described precisely, because the claim it supports is narrower than it is often made to carry. The primary endpoint was safety and tolerability, not efficacy; the improvement in forced vital capacity observed in the higher-dose arm, against a decline on placebo, was a secondary and encouraging signal rather than a prespecified demonstration of benefit. That the indication is fibrotic rather than oncological does not diminish its significance for the present argument. What it establishes is a randomised clinical proof of concept: that an AI-enabled workflow spanning target identification and generative molecular design can carry a compound into a controlled human trial and produce an encouraging efficacy signal there. That is a genuine milestone, and it is not the same thing as comprehensive clinical validation of AI-driven drug discovery, which a phase 2a study of this size could not provide.

### 7.2. Success Rates and the Meaning of Attrition

These individual proofs must be set against systematic evidence on how artificial-intelligence-discovered drugs fare in aggregate, and the most informative such analysis examined the clinical progression of molecules from artificial-intelligence-native companies. Its findings are double-edged in a way that is highly diagnostic. In phase 1, artificial-intelligence-discovered molecules succeeded at a rate of roughly eighty to ninety per cent, substantially above the historical industry average, whereas in phase 2 the success rate fell to approximately forty per cent, comparable to historical norms, albeit on a still-limited sample [144]. The interpretation is illuminating and sobering at once. Phase 1 chiefly tests safety and pharmacokinetics properties of the molecule and the elevated success rate suggests that generative and predictive methods are indeed good at producing well-behaved, drug-like molecules with favourable early profiles. Phase 2 tests efficacy, which is a property of the target hypothesis and the underlying biology, and the reversion to historical rates indicates that artificial intelligence does not yet de-risk the biological question of whether engaging a given target produces clinical benefit.

These figures deserve corresponding care, because the dataset behind them is small and not randomly sampled. The analysis identified on the order of sixty molecules in clinical development from artificial-intelligence-native companies against a total industry pipeline of several thousand, and its authors flag the limits themselves: few programmes have completed the transitions being counted, disclosure is voluntary and favours success, the therapeutic-area mix is not representative, and firms of this kind have tended to pursue well-validated targets, which lowers biological risk for reasons unconnected to how the molecule was designed. The definition of an artificial-intelligence-discovered drug is itself elastic, spanning everything from a learned property filter applied late in optimisation to a fully generative campaign. The evidence is therefore consistent with the proposition that these methods improve early-stage molecular optimisation; it does not establish it causally, and it establishes nothing at all about efficacy. We report the pattern because it is the best available and the interpretation because it is the most natural, while noting that both could shift substantially as the sample matures.

For the undruggable oncology targets, this distinction is exactly the relevant one. The achievement of the methods surveyed here is overwhelmingly at the level of the molecule and its interaction with a structure: finding a cryptic pocket, designing a ligand that binds it with predicted affinity, optimising selectivity and properties. Whether engaging that target then yields durable clinical benefit in the face of tumour heterogeneity, adaptive signalling and acquired resistance is a question that the historical attrition statistics, against which the long-run success rate of all oncology programmes is among the lowest of any therapeutic area [145], suggest will not be answered by chemistry alone. The realistic reading is that artificial intelligence is a powerful accelerator of the discovery and optimisation of chemical matter, capable now of closing the design loop even in the clinic, but that it operates on, rather than replaces, the biological validation that ultimately governs success.

### 7.3. Resistance and the Moving Target

A specific translational reality deserves emphasis because it is intrinsic to oncology and conditions the value of any single-target success: resistance. The clinical history of the KRAS-G12C inhibitors, the very compounds that vindicated the undruggable enterprise, already includes the emergence of acquired resistance through secondary mutations in KRAS that prevent inhibitor binding, through reactivation of the pathway by upstream or parallel signalling, and through other adaptations, so that initial responses are frequently followed by relapse [146,147]. This is the biological rationale for the combination strategies discussed in Section 5.4, and it reframes the design objective: against an adaptive disease, the goal is not a single optimal molecule but a regimen, and the network-directed and polypharmacological logic of Section 6.5 becomes not a refinement but a necessity. Artificial-intelligence methods capable of reasoning over combinations and over the evolutionary trajectory of resistance, rather than over single binding events, are correspondingly the ones whose development matters most for durable benefit.

The mechanisms are now characterised in some detail. In the first systematic analysis of patients progressing on adagrasib, putative resistance mechanisms were identified in a substantial minority of the cohort, frequently more than one in the same patient: secondary mutations within the switch-II pocket itself, at residues including Tyr96, His95 and Arg68, which sterically or electronically prevent inhibitor binding while preserving the function of the protein; second-site mutations at codons 12 and 13 arising in trans, which convert the tumour to a KRAS allele the drug does not recognise; and bypass events including MET amplification, oncogenic fusions and mitogen-activated protein kinase pathway alterations that restore signalling downstream of the inhibited protein [146]. A distinct switch-II substitution, Tyr96 to aspartate, was shown to contract the pocket and to defeat both clinical G12C inhibitors [147]. The picture is one of convergent escape by multiple independent routes, which is the signature of strong selective pressure applied to a single node.

Stated that way, the problem is not purely biological, and it is worth being explicit about what artificial intelligence can contribute, since a review that catalogues computational capability should not fall silent precisely where the clinical difficulty lies. Four contributions are available in principle, and the first two are already practised.

The first is anticipation rather than reaction. The set of mutations that could confer resistance to a given inhibitor is enumerable: the residues lining the pocket are known, and the substitutions reachable by a single nucleotide change are finite. The effect of each on inhibitor binding can therefore be computed prospectively, by the same alchemical free-energy methods used to rank ligands in Section 4.2, as a scan of the change in binding free energy over candidate mutations, and cross-checked against the constraint that the mutant must remain catalytically and structurally functional if the tumour is to survive it. Combining a predicted loss of inhibitor affinity with a predicted retention of function and with the mutational accessibility of the codon yields a ranked forecast of escape routes before the first patient is dosed, and with it a targeted assay panel and a circulating-tumour-DNA panel. Deep mutational scanning supplies the experimental counterpart against which such forecasts are calibrated, and the machine-learned variant-effect models discussed in Section 3.4 supply a cheap prior over functional tolerance [80].

The second is designing against that forecast rather than against the wild-type structure alone. Once the probable escape mutants are known, they become additional receptors in the design problem: a generative campaign can be conditioned on an ensemble of pocket variants and scored on its worst case rather than its mean, an objective that systematically favours chemotypes whose binding does not depend critically on the residues most likely to mutate. This is the molecular-design expression of the logic that produced the mutation-agnostic strategy in practice: the tri-complex pan-RAS(ON) agents engage a surface common to RAS proteins in the active state rather than a mutation-specific feature, and the randomised survival benefit reported for daraxonrasib in previously treated metastatic pancreatic cancer, extending across both RAS-mutant and RAS wild-type disease, is the clinical evidence that breadth of this kind can be bought without an unacceptable loss of therapeutic index [24].

The third is combination selection, which is a prediction problem over pathways rather than over binding sites. Bypass resistance is by definition a network phenomenon, and the data needed to model it already exist: genome-scale loss-of-function screens across hundreds of cancer cell lines define the dependency structure into which an inhibitor is introduced, and machine-learned models of synthetic lethality and of adaptive pathway rewiring can nominate the partner most likely to close the escape route that a given tumour context favours. Section 6.5 develops the polypharmacological logic in general terms; the point to register here is that the choice of combination is a computational problem currently under-addressed relative to the choice of molecule [139].

The fourth, and by some distance the least mature, is temporal. Resistance emerges under a dosing schedule, and circulating tumour DNA now makes that emergence observable before it is radiographically manifest. Models that treat therapy as a sequential decision problem in which the objective is the expected duration of disease control under an evolving tumour rather than the potency of a single agent, and in which observed subclonal dynamics update the policy are an established idea in mathematical oncology and an almost entirely unexploited application of the apparatus assembled in this review. The design objective, stated plainly, should be a regimen and a schedule rather than a molecule. The methods described in Section 2 and Section 4 optimise the molecule superbly and the regimen not at all, and closing that gap is where artificial intelligence would most change what happens to a patient.

One further point belongs here, because it generalises the argument beyond KRAS and has become visible only recently. Acquired resistance has now been characterised for p53-Y220C reactivation as well. Profiling of circulating tumour DNA, tumour biopsies and rapid-autopsy specimens from patients progressing on rezatapopt identified multiple heterogeneous secondary TP53 alterations arising in cis with Y220C: mutations in the DNA-binding domain, together with frameshift and nonsense changes, that abolish transcriptional activity outright; and substitutions within the Y220C crevice itself that are predicted to obstruct drug binding [148]. The second class is the exact analogue of the switch-II pocket mutations that defeat the KRAS-G12C inhibitors, and it is therefore open to the same computational treatment: the crevice is small, its lining residues are known, and the substitutions reachable by a single nucleotide change are enumerable, so the prospective free-energy scan described above applies directly.

The first class is more troubling, and the asymmetry deserves to be stated plainly, because it has no counterpart in oncogene inhibition. A tumour escaping an oncogene inhibitor must preserve the function of the oncogene while evading the drug, which sharply constrains the mutations available to it and is the reason the KRAS escape routes of Section 7.3 are few enough to enumerate. A tumour escaping the restoration of a tumour suppressor faces no such constraint: any second alteration that destroys p53 function will serve, and the evolutionary escape space is correspondingly larger. Conformational stabilisation may therefore prove intrinsically more fragile than oncogene blockade. That is a consideration for how such agents are combined and sequenced, and for how early resistance is monitored, rather than an argument against the modality; but it qualifies the optimism with which the p53 case is usually presented, including in Section 5.2.

## 8. Safety, Dual Use, and Governance

The same generative methods that design beneficial molecules are, by their nature, indifferent to the sign of the objective they optimise, and this symmetry carries a serious dual-use hazard that the field has begun to confront explicitly. In a now-widely discussed demonstration, a group adapted a generative model ordinarily used to design drugs by inverting its scoring so as to reward, rather than penalise, predicted toxicity; in under six hours of computation the system generated tens of thousands of candidate toxic molecules, including not only known chemical-warfare agents but novel structures predicted to exceed them in toxicity [149]. The molecules were never synthesised, and the authors published the result precisely as a warning rather than a recipe, withholding operational detail; but the demonstration establishes something narrower and still serious: that a molecular-generation framework built for therapeutic design can be redirected, with little more than a change in sign in its objective, toward a goal that rewards predicted toxicity. What was shown is the ease of that redirection and the fertility of the resulting search, not the toxicity of the particular structures produced, none of which was synthesised or experimentally tested. The dual-use hazard lies in the first of these and does not depend on the second.

The implications for governance are still being worked out, but several follow directly. The expertise, software and data required to run such generative models are increasingly widespread, so that the safeguards cannot rest on scarcity alone; responsible deployment plausibly requires a combination of technical controls within the design software, restrictions on the most dangerous training data and synthesis capabilities, and norms of disclosure of the kind the toxicity demonstration itself exemplified. For a review concerned with drugging the undruggable, the dual-use problem is not a peripheral ethical addendum but an intrinsic property of the central tool: the more capable generative design becomes at finding molecules that bind difficult targets, the more capable it becomes at finding molecules that do harm, and the governance of the technology must mature in step with its power.

## 9. Discussion: The Shape of an Integrated Pipeline

Drawing the threads together, the picture that emerges is not of a single decisive technology but of an integrated pipeline in which several advances, each individually insufficient, combine to render the undruggable approachable. The pipeline begins with structure and ensemble: the prediction of a target’s structure, increasingly of its complexes, and the characterisation of its conformational ensemble by molecular dynamics, from which the cryptic and allosteric pockets that constitute the actual points of attack are identified, now with the assistance of dedicated machine-learning predictors. It proceeds to design: the generation of candidate molecules conditioned on the identified pocket, by the equivariant, diffusion-based and flow-based generative models whose mathematics occupied Section 2, with the modality covalent, allosteric, glue, degrader chosen to match the biophysics of the target. And it closes with evaluation: the ranking of candidates by physics-based free-energy methods at near-chemical accuracy, the filtering of physically invalid proposals, the synthesis-aware constraints and retrosynthetic planning set out in Section 2.6, and ultimately experimental and clinical testing. Each stage has a characteristic strength and a characteristic failure mode, and the competence of the whole is limited by its weakest link. Figure 1 renders that pipeline as a whole, from the genomic layer at which a target is chosen to the clinical layer at which the resulting molecule is tested, together with the two feedback loops the design–make–test–analyse cycle and the return of clinical resistance data as a new target state that make it iterative rather than linear.

It is therefore essential to state plainly what is proven and what remains open. On the side of the proven: that cryptic and allosteric pockets exist on canonical undruggable targets and can be discovered and exploited, with KRAS the definitive example; that covalent, allosteric and conformational-stabilisation modalities can convert specific intractable proteins into sources of approved or clinically active drugs, as KRAS, p53-Y220C and SHP2 attest; that structure prediction now achieves very high accuracy for many well-folded single-chain domains and has advanced substantially for complexes, while conformational ensembles, disordered and cryptic states and ligand-induced conformations remain considerably harder; that physics-based free-energy methods reach roughly chemical accuracy in favourable, congeneric regimes; and that generative models can produce real, active, novel chemical matter, now validated as far as a positive controlled clinical trial in a non-oncological indication. On the side of the open: that the structure-based generative design of small molecules against a genuinely novel undruggable oncology target, carried prospectively to the clinic, has not yet been demonstrated; that among the most valuable modalities for several of these targets, the molecular glue remains largely beyond the reach of de novo computational design and is the frontier we particularly flag; that docking and pose prediction by current deep-learning methods still fail tests of physical validity that classical physics-based methods pass; that free-energy accuracy degrades outside congeneric series and is often limited by force-field quality; that the synthesizability of generated matter, while no longer the unanswerable objection it once was, given the constrained-generation and retrosynthetic methods set out in Section 2.6, still has to be engineered into a pipeline deliberately and is not obtained by default; and that the biological questions of efficacy and resistance, which dominate clinical attrition, are not addressed by molecular design at all. The honest synthesis is that artificial intelligence and physics-based computation have decisively expanded the druggable proteome and accelerated the discovery of chemical matter, while the translation of that chemical matter into durable clinical benefit against cancer remains governed by a biology that the methods illuminate but do not yet control.

Several directions follow from this assessment as the natural near-term agenda. The integration of ensemble-aware generative design with rigorous free-energy evaluation, so that molecules are generated against, and scored on, conformational distributions rather than single structures, addresses the disordered and cryptic-pocket targets most directly. The extension of generative methods to the ternary and induced-proximity modalities would open the glue and degrader space to rational design and is, in our view, the single most valuable methodological advance the field could make for undruggable oncology. And the coupling of molecular design to models of resistance and combination, so that the object of optimisation becomes a regimen robust to tumour evolution rather than an isolated binder, aligns the technology with the actual clinical problem. None of these is beyond the trajectory of current research, and each would convert a present limitation into a capability.

## 10. Conclusions

The category of the undruggable was never a statement about the importance of the proteins it contained, which includes the most central drivers of human cancer, but about the methods available to attack them; and the central claim of this review is that the category has contracted, and continues to contract, because the methods have changed. The change is the convergence of three developments: a revised understanding of the binding site as a dynamic, sometimes cryptic feature of a conformational ensemble rather than a fixed cavity; the maturation of physics-based and machine-learned methods that read binding from three-dimensional structure with increasing accuracy; and the advent of generative artificial intelligence that designs molecules conditioned directly on a target. KRAS, once the emblem of futility, is now the emblem of what these methods together can achieve, and p53, MYC and SHP2 mark successive points along a frontier that is still advancing.

Yet the same survey that documents these advances counsels precision about their limits. The achievements are concentrated at the level of the molecule and its engagement with a structure, where artificial intelligence has proven a genuine and accelerating force; the persistent difficulties lie at the level of biology efficacy, heterogeneity, resistance where clinical attrition remains governed by factors that molecular design does not yet reach, and at the level of the hardest modalities, where the molecular glue in particular awaits a generative method equal to its promise. The realistic and, we would argue, the genuinely exciting conclusion is neither the triumphalism that declares drug discovery solved nor the scepticism that dismisses the advances as hype, but the recognition that a powerful new instrument has been added to a difficult enterprise: artificial intelligence has made the undruggable approachable, and the task now is the patient, biologically grounded work of converting approachability into medicines.

## Figures and Tables

**Table 1:** The four undruggable targets: biological hurdle, failure of classical approaches, computational contribution, and resulting chemical matter. In the fourth column, the parenthetical numerals denote the three categories set out in Section 1.3: (1) experimental, structural and classical computational work, which in every case did the decisive work; (2) demonstrated contributions of machine learning; (3) prospective roles for generative design, not yet realised in any approved or clinically successful agent against these targets. Clinical status is as described in Section 7 and in the sources cited there; agents in active development should be checked against current trial records.

Target	Biological Basis of Undruggability	Why Classical Approaches Failed	Computational Contribution, by Category	Chemical Matter and Clinical Status
KRAS	A GTPase cycling between GDP- and GTP-bound states, binding nucleotide with picomolar to femtomolar affinity, with a smooth effector interface and no deep pocket outside the nucleotide site.	Any orthosteric ligand must outcompete millimolar cellular GTP, leaving no thermodynamic foothold, and the switch-II pocket is invisible in ground-state structures.	(1) Molecular dynamics and enhanced sampling that exposed the cryptic switch-II pocket; crystallography-guided covalent and non-covalent structure-based optimisation; covalent kinetics; tri-complex design against the active state. (2) Machine-learned cryptic-pocket prediction, validated against KRAS after the fact rather than constituting its discovery route. (3) Generative design conditioned on ensembles of pocket variants, proposed in Section 7.3 and not yet the origin of any clinical KRAS molecule.	Approved: sotorasib and adagrasib for G12C, and sotorasib with panitumumab for G12C colorectal cancer; daraxonrasib (RMC-6236), a pan-RAS(ON) agent, approved in August 2026 for previously treated metastatic pancreatic adenocarcinoma, with an indication not restricted by RAS allele. Discontinued: MRTX1133, the first clinical G12D inhibitor, whose phase 1 study was terminated; it remains a chemical proof of principle rather than an active programme.
p53	A tumour suppressor lost by mutation, so that the therapeutic aim is restoration of function rather than inhibition; the mutant DNA-binding domain is thermodynamically destabilised.	There is nothing to block and no pocket in the wild-type fold, and restoring a fold was never a classical medicinal-chemistry objective.	(1) Structural characterisation of the mutation-created crevice; conventional in silico screening against it; molecular dynamics of its transient character; conformational-stabilisation design taking thermal stabilisation as the objective. (2) No decisive machine-learning contribution to the campaign that produced the clinical candidate. (3) Ensemble-conditioned generative design of stabilisers, an open opportunity.	PhiKan and related probes; rezatapopt (PC14586), with a published phase 1 objective response rate of twenty per cent across tumour types, and a registrational phase 2 basket trial reporting an interim ovarian-cohort response rate of roughly forty-four per cent, not yet published in full; MDM2 antagonists of the nutlin class where p53 is retained as wild type.
MYC	An intrinsically disordered basic helix–loop–helix leucine zipper acting through an extended interface with MAX, with no folded pocket anywhere on the functional surface.	There is no cavity to fill; the target is a fluctuating ensemble rather than a structure, and nuclear localisation and rapid turnover complicate exposure.	(1) Ensemble characterisation by molecular dynamics; interface and hot-spot analysis; protein engineering, which produced the clinical agent. (2) Limited. (3) Ensemble-aware generative small-molecule design, the plausible eventual route and as yet undelivered. Omomyc is an achievement of protein engineering, not an AI-designed small molecule.	Omomyc, delivered as OMO-103, has completed a phase 1 trial; direct small-molecule inhibitors remain preclinical.
SHP2	A protein tyrosine phosphatase whose catalytic site is shallow, highly charged and closely conserved across a large paralogous family.	Active-site binders are polar, cell-impermeant and non-selective, and paralogue selectivity is unattainable at the catalytic site itself.	(1) Structure-based discovery of the allosteric tunnel at the three-domain interface; molecular dynamics of the autoinhibited-to-open transition; classical allosteric medicinal chemistry. (2) Machine-learned modelling of combination and resistance behaviour. (3) Generative design against allosteric sites, largely prospective.	SHP099 as the defining tool compound; TNO155 and congeners in clinical trials, largely in combination with KRAS-G12C and other pathway inhibitors.

**Table 2:** Sources, search strategy and eligibility criteria for the literature underpinning this review.

Element	Specification
Bibliographic databases	PubMed/MEDLINE, Scopus, Web of Science Core Collection.
Preprint servers and machine-learning venues	arXiv, bioRxiv, ChemRxiv; proceedings of NeurIPS, ICML and ICLR, in which a substantial part of the methodological literature of this field is published and never appears in journals.
Clinical and regulatory sources	ClinicalTrials.gov for trial status; AACR, ASCO and ESMO abstract archives for results not yet in full publication.
Coverage	1 January 2013 to 31 May 2026; last updated May 2026. No lower date limit was applied to landmark methodological and biological papers reached by backward citation chaining. Clinical, regulatory and bibliographic status refreshed through 31 August 2026.
Block 1—targets	“undruggable” OR “intractable target” OR KRAS OR RAS OR TP53 OR p53 OR Y220C OR MYC OR SHP2 OR PTPN11 OR “transcription factor” OR “protein–protein interaction” OR “intrinsically disordered protein”
Block 2—methods	“machine learning” OR “deep learning” OR “artificial intelligence” OR “generative model” OR “diffusion model” OR “equivariant neural network” OR GFlowNet OR docking OR “virtual screening” OR “free energy perturbation” OR “molecular dynamics” OR “cryptic pocket” OR AlphaFold
Block 3—modalities	“covalent inhibitor” OR allosteric OR “molecular glue” OR PROTAC OR degrader OR “targeted protein degradation” OR polypharmacology
Combination	Block 1 AND Block 2, with Block 3 applied as an additional AND for the modality sections. Terms within a block were combined with OR.
Inclusion criteria	Primary methodological papers; structural, biophysical and biochemical studies; clinical reports. Reviews were used for context rather than as evidence.
Exclusion criteria	Non-English records; abstracts reporting no data; preprints for which a peer-reviewed version exists. Preprints are cited only where no peer-reviewed version was available and are marked as such in the reference list.
Stated limitations	This is a narrative and not a systematic review. There was no PRISMA flow diagram, no dual independent screening and no formal risk-of-bias assessment. Selection of material is therefore interpretive, which is why the text is explicit throughout about where evidence is strong, where it is preliminary and where it is contested.

**Table 3:** Targets considered for detailed treatment, the decision taken in each case, and the grounds for it.

Target or Class	Status in This Review	Grounds
KRAS	In depth (Section 5.1)	Overwhelming substrate competition combined with a pocket absent at rest; the defining case of a cryptic site exploited covalently.
p53	In depth (Section 5.2)	A loss of function to be restored rather than a gain to be blocked, and a cavity created by the mutation itself; the defining case of conformational stabilisation.
MYC	In depth (Section 5.3)	An extended, intrinsically disordered interface with no folded pocket at all; the defining case of designing against an ensemble.
SHP2	In depth (Section 5.4)	A catalytic site too polar and too conserved to drug selectively; the defining case of an allosteric solution remote from the active site.
NRAS, HRAS	Within Section 5.1	Share the switch architecture, nucleotide affinity and effector interface of KRAS, differing chiefly in hypervariable region, membrane anchoring and mutational spectrum. Separate treatment would duplicate the argument.
STAT3	Generalisation in Section 5.5	Shares the transcription-factor and interface problem with MYC. Its specific difficulty is paralogue selectivity within the STAT family, which parallels the phosphatase argument of Section 5.4.
β-catenin and WNT effectors	Generalisation in Section 5.5	An armadillo-repeat interface of the dimensions that make protein–protein interactions hard, with the added constraint that the same surface serves physiological partners.
Oncogenic fusion proteins	Generalisation in Section 5.5	Heterogeneous: those with druggable enzymatic domains are conventional, while those acting through disorder inherit the MYC analysis.
BCL-2 family	Excluded	A solved rather than an open problem by the standard used here. The fragment-based campaign that produced venetoclax predates the generative era and is better read as the precedent establishing that interfaces contain druggable hot spots.
Epigenetic readers and writers	Excluded	Possess well-formed, conventionally druggable pockets. Their difficulties have been selectivity, on-target toxicity and therapeutic index rather than tractability; they were never members of the undruggable class as defined in Section 1.1.

**Table 4:** A reader’s map of Section 2: each formalism, the drug-design question it exists to answer, and the way in which it characteristically fails.

Formalism	The Question It Exists to Answer	Section	Characteristic Failure Mode
Molecular representations: strings, graphs, three-dimensional point clouds	How is a molecule presented to a model without discarding the geometry that decides whether it fits a cavity?	Section 2.1	Strings and graphs discard three-dimensional information; point clouds restore it but demand explicit handling of symmetry.
E(3)/SE(3)-equivariant graph networks	How can a model reason in three dimensions without having to learn from data that physics is indifferent to rotation and translation?	Section 2.2	Expressivity traded against cost. Equivariance guarantees correct geometry, not correct chemistry.
Denoising diffusion and score-based models	How are genuinely novel molecules generated, conditioned on one specific pocket rather than selected from a library?	Section 2.3	Samples may be strained, physically invalid, or impossible to synthesise.
Generative flow networks	How is a diverse set of good candidates obtained rather than a single optimum, given that any one chemotype usually fails for reasons unrelated to binding?	Section 2.4	Any mis-specification of the reward propagates directly into the sampled distribution.
Docking and machine-learned scoring	How are millions or billions of proposals triaged at a cost that makes the campaign feasible?	Section 2.5	A plausible pose is not a physically valid one, and benchmark performance is systematically inflated.
Synthesis-aware generation and retrosynthetic scoring	Can the molecule that has been proposed actually be made, and at what cost?	Section 2.6	Accessibility scores are proxies for the output of one planner rather than for success in a laboratory.

**Table 6:** The six technical barriers to de novo computational design of molecular glues, and the computational responses now emerging against each.

Technical Barrier	Why It Defeats Current Methods	Emerging Computational Response
No pocket on which to condition	Structure-based generative models take a receptor cavity as input. On the target side of a glue complex, that cavity does not exist before the compound arrives, and the site occupied on the presenter is often not a recognisable pocket either. The conditioning signal the method requires is simply absent.	Interface-directed rather than pocket-directed conditioning; generation guided by a predicted ternary interface rather than by an apo cavity.
A three-body problem with two mobile protein surfaces	Binary docking has six rigid-body degrees of freedom plus ligand torsions; a ternary complex adds the full relative orientation of two proteins, each with its own induced fit. Benchmarks on post-training-cut-off structures show current co-folding models recovering roughly a third of glue–protein interactions, with apparent accuracy inflated by accessory subunits.	Co-folding architectures adapted specifically to ternary prediction; steered and guided sampling; benchmark sets restricted to glue and degrader complexes so that progress can be measured rather than asserted.
Cooperativity is an interface free energy	The quantity to be optimised is a difference in free energies across a large, solvated protein–protein interface dominated by desolvation and ordered water. The available scoring functions were parameterised on buried, drug-like cavities, so the error bar on a computed cooperativity is of the same order as the effect being designed for.	Explicit-solvent treatment of interfacial water; interface-specific reparameterisation; use of computed cooperativity for ranking within a series rather than as an absolute prediction.
The objective is non-monotonic	Degradation is a multi-step catalytic process ternary assembly, positioning of an accessible lysine, processive ubiquitin transfer, proteasomal engagement, catalytic turnover and is not a monotonic function of ternary affinity. Excess compound saturates the partners separately and dismantles the complex.	Objectives defined on simulated degradation rather than on affinity; kinetic models of the ubiquitination cascade used as the reward; explicit penalisation of the hook regime.
Scarce and systematically biased data	Experimentally determined glue ternary complexes number in the low hundreds in total, and in the tens once restricted to structures released after model training, and are drawn overwhelmingly from cereblon and DCAF15. Only a small minority of the several hundred human E3 ligases have been productively liganded at all.	Chemical-genetic and degradation-proteomic discovery used to generate training data rather than to replace design; transfer from general protein–protein interface data; ligase-stratified reporting of performance.
Discontinuous structure-activity relationships	Because activity depends on complementarity across an induced interface rather than on occupancy of a defined cavity, minor chemical changes can create or abolish degradation outright, so the smooth local landscapes exploited by gradient-based and reinforcement-learning optimisers are not reliably present.	Sampling methods tolerant of rugged landscapes, including the flow-network approaches of Section 2.4; active learning with an experimental degradation readout in the loop.

## Data Availability

No new data were created or analyzed in this study. Data sharing is not applicable to this article.

## Abbreviations

ADMET, absorption-distribution-metabolism-excretion-toxicity; AF, AlphaFold; CADD, computer-aided drug design; CASP, computer-aided synthesis planning; ctDNA, circulating tumour DNA; DEL, DNA-encoded library; DMS, deep mutational scanning; DMTA, design–make–test–analyse; E3, E3 ubiquitin ligase; EGNN, E(n)-equivariant graph neural network; FEP, free-energy perturbation; GDP/GTP, guanosine di-/tri-phosphate; GFlowNet, generative flow network; GNN, graph neural network; IDP, intrinsically disordered protein; MD, molecular dynamics; MM-PBSA, molecular-mechanics Poisson–Boltzmann surface area; MoA, mechanism of action; PK/PD, pharmacokinetics/pharmacodynamics; PPI, protein–protein interaction; PROTAC, proteolysis-targeting chimaera; RBFE, relative binding free energy; RL, reinforcement learning; RMSD, root-mean-square deviation; RTK, receptor tyrosine kinase; SA score, synthetic accessibility score; SBDD, structure-based drug design; SDE, stochastic differential equation; SII-P, switch-II pocket; TF, transcription factor; TI, thermodynamic integration; TPD, targeted protein degradation.
